# Multiclass Assays for Measuring Environmental Chemical Mixture Exposure: Analytical Methodologies and Applications in Exposomics Research

**DOI:** 10.3390/metabo15110742

**Published:** 2025-11-16

**Authors:** Ravikumar Jagani, Jasmin Chovatiya, Divya Pulivarthi, Anil K. Meher, Dhavalkumar Patel, Hiraj Patel, Sandipkumar Teraiya, Syam S. Andra

**Affiliations:** Institute for Exposomic Research, Department of Environmental Medicine, Icahn School of Medicine at Mount Sinai, New York, NY 10029, USA

**Keywords:** biomonitoring, chemical exposome, environmental contaminants, exposome-wide association studies, LC-HRMS, LC-MS/MS, matrix effects, multiclass assays, multiple reaction monitoring, solid phase extraction

## Abstract

Background/Objectives: The exposome includes all environmental exposures throughout a lifetime and profoundly influences health and disease, reflecting the totality of environmental chemical exposures throughout an individual’s life, encompassing both natural and anthropogenic chemicals from external sources. Conventional methods for environmental chemical analysis have generally concentrated on individual representatives or substance classes; however, single analyte/class techniques are impractical for extensive epidemiological studies that require the analysis of thousands of samples, as anticipated for forthcoming exposome-wide association studies. This narrative review analyzes the evolution and implementation of multiclass assays for measuring ambient chemical exposure, emphasizing analytical techniques that provide the concurrent quantification of various chemical classes. Methods: This narrative review consolidates existing literature on multiclass analytical methodologies for measuring exposure to environmental chemical mixtures, encompassing mass spectrometry platforms, sample preparation techniques, chromatographic separation methods, and validation strategies for thorough exposure assessment in human biomonitoring research. The review includes liquid chromatography–mass spectrometry techniques, solid-phase extraction methods, and data analysis strategies for both targeted and non-targeted study. Results: Multi-class methodologies provide the concurrent quantification of compounds from many classes without the necessity for distinct conventional procedures, thus minimizing time, expense, and sample volume. The robustness of the method indicates appropriate extraction recovery and matrix effects between 60 and 130%, inter-/intra-day precision under 30%, and remarkable sensitivity with detection limits from 0.015 to 50 pg/mL for 60–80% of analytes in the examined human matrices. The methodology facilitates the concurrent identification of the endogenous metabolome, food-associated metabolites, medicines, home chemicals, environmental contaminants, and microbiota derivatives, including over 1000 chemicals and metabolites in total. Conclusions: These thorough analytical methods deliver the requisite performance for extensive exposome-wide association studies, yielding quantitative results and uncovering unforeseen exposures, thereby augmenting our comprehension of the chemical exposome, which is essential for advancing disease prevention in public health and personalized medicine.

## 1. Introduction

### 1.1. The Exposome Paradigm and Environmental Health

**Figure 1:**
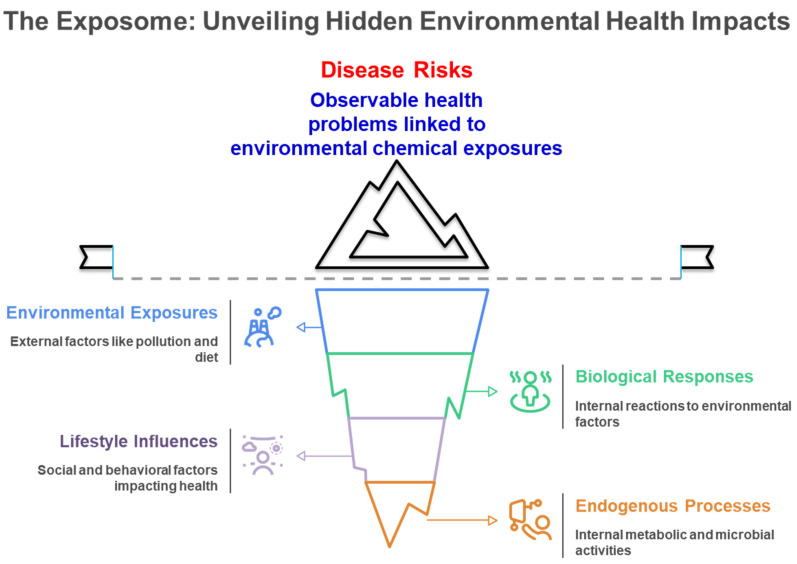
Conceptual Framework of the Exposome and Its Health Impacts.

**Figure 2:**
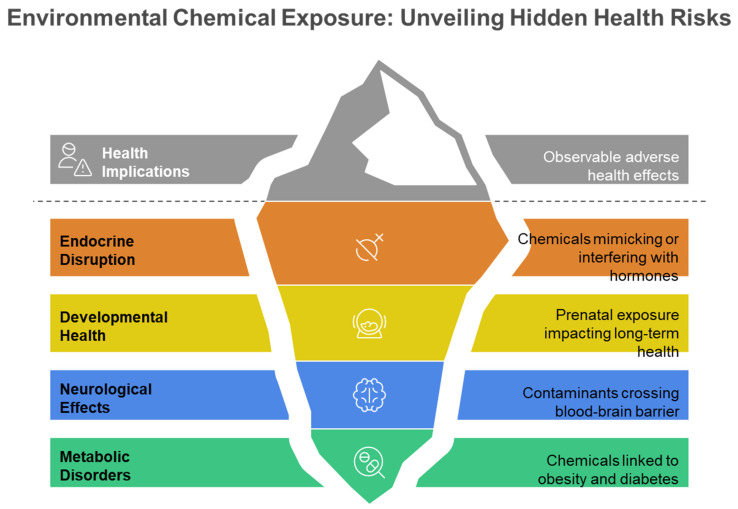
Schematic representation of major health implications associated with environmental chemical exposures.

The exposome signifies a transformative approach in environmental health research, including the entirety of environmental exposures experienced by an individual over their lifetime and their significant influence on human health and disease [1,2,3,4,5]. The exposome paradigm was initially proposed by Wild in 2005 [2] and is defined as the aggregate of exposures that individual encounters during their lifetime [1,2,3,4,5,6]. Figure 1 illustrates the comprehensive nature of the exposome paradigm, demonstrating how external environmental exposures, lifestyle influences, and endogenous processes interact to produce biological responses that ultimately manifest as observable disease risks, representing the cumulative environmental influences on human health throughout the lifespan. The chemical exposome covers the totality of environmental chemical exposures experienced by an individual over their lifetime, incorporating both natural and anthropogenic chemicals from external sources—such as inhalation of polluted air, the intake of food compounds and medications, along with the consumption of tainted food and water—as well as internal exposure sources, including metabolic byproducts from gut microbiota (Figure 1) [3,6,7]. Environmental factors significantly influence health status, surpassing the previously acknowledged impact of the intrinsic genome. Factors such as individual food, smoking, and air pollution may account for approximately 46% of global mortality [8,9]. Environmental exposure has emerged as a significant risk factor for public health, correlating with unknown illness risks such as cardiovascular disease, cancer, respiratory disorders, and other chronic conditions [5,6,10]. Figure 2 depicts the varied health consequences of environmental chemical exposures, highlighting the role of the chemical exposome in multiple disease pathways, such as endocrine disruption, developmental disorders, neurological effects, and metabolic dysfunction throughout the human lifespan. These diverse health endpoints reflect the complex toxicological effects of the chemical exposome on human health. This comprehensive understanding of environmental influences has driven the need for more sophisticated analytical approaches capable of capturing the complexity of human chemical exposures.

### 1.2. Evolution of Multiclass Analytical Approaches

**Figure 3:**
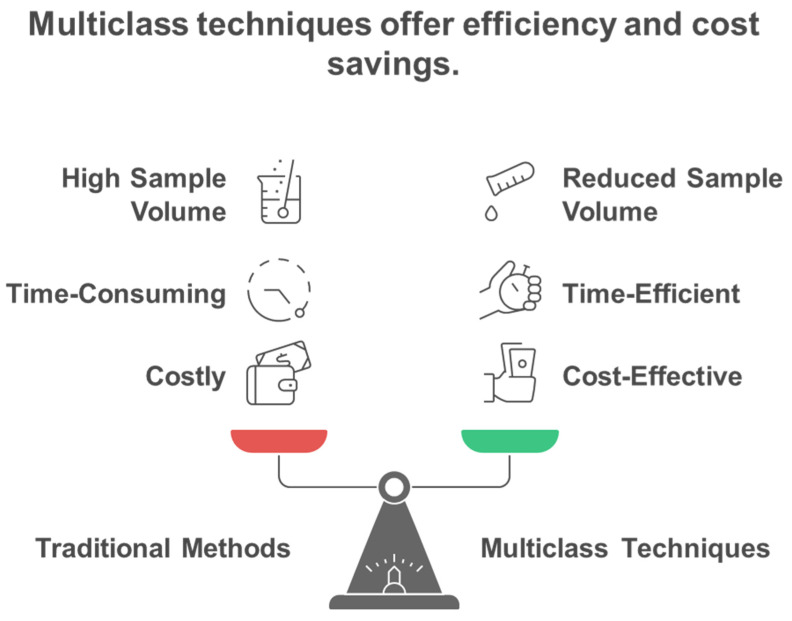
Comparison of traditional single class versus multiclass analytical approaches for environmental chemical exposure measurement.

**Figure 4:**
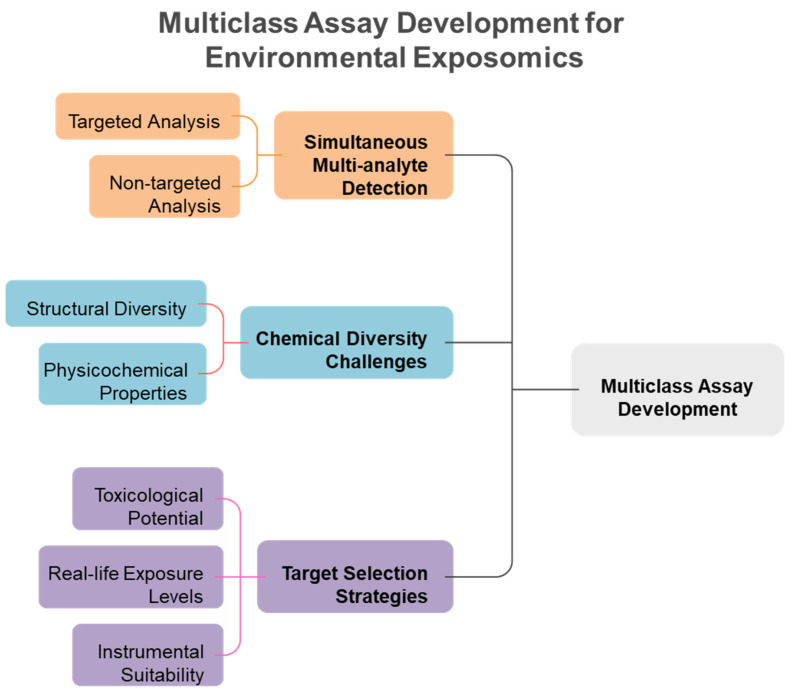
Schematic overview of the key components and challenges in developing multiclass analytical methods for comprehensive exposome characterization.

Traditional single-analyte approaches have proven inadequate for comprehensive exposome measurement, leading to the development of innovative multiclass analytical methodologies that can simultaneously quantify diverse chemical classes. Conventional methods for analyzing environmental chemicals have generally concentrated on individual representatives or specific substance classes. Consequently, this focus may lead to the banning of certain chemicals, only to be replaced by less studied analogs that could potentially exhibit similar or even more severe toxicological effects [11]. Methods focusing on a single analyte or class are not suitable for extensive epidemiological studies that require the analysis of thousands of samples, as suggested for upcoming exposome-wide association studies [11]. Most targeted analytical methods quantify fewer than 15 biomarkers of exposure from a singular chemical class within each biospecimen, employing class-specific extractions and instrumental analyses for substances such as dialkyl phosphates (DAPs), EP, herbicides, OPFR, OP pesticides, PAH, PHTH, pyrethroids, tobacco smoke, and VOC [12]. To address these challenges, multi-class techniques are becoming increasingly favored by utilizing extractions that enhance various classes of chemicals in human specimens and enabling simultaneous detection. This approach allows for the measurement of multiple classes of chemicals without the need for separate conventional workflows, ultimately leading to reductions in time, cost, and sample volume [12,13]. Figure 3 illustrates the fundamental advantage of multiclass analytical approaches over traditional single-class methods, demonstrating how simultaneous measurement of diverse chemical classes achieves significant improvements in analytical efficiency, cost-effectiveness, and sample conservation for large-scale exposome studies. Traditional approaches require separate analytical workflows for each chemical class, creating significant bottlenecks in large-scale studies. Multi-class techniques overcome these limitations by enabling simultaneous detection of diverse chemical classes without separate conventional workflows, providing substantial reductions in analysis time, cost, and required sample volumes while maintaining comprehensive chemical coverage. The shift towards multiclass analytical methods has been motivated by the necessity to tackle the intricacies of the exposome, where food-derived metabolites and endogenous compounds typically exist in the millimolar to picomolar concentration range, whereas pollutants may be detected at levels three orders of magnitude lower [14,15]. Recent advancements in analytical instrumentation have accelerated the detection of trace amounts of xenobiotics in human tissues and biofluids, enabling a more accurate quantitative evaluation of an individual’s chemical burden [16]. However, analytical challenges remain, including matrix effects, sensitivity issues, and the need for non-discriminatory sample preparation methods that balance interference reduction with broad chemical coverage [17,18]. The recent technological advancements allow for the concurrent analysis of numerous compounds spanning various chemical classes, transforming the capabilities of exposome research (Table 1). Figure 4 illustrates the comprehensive framework for multiclass assay development in exposomics research, highlighting the integration of targeted and non-targeted approaches while addressing the fundamental challenges posed by chemical diversity in environmental exposure assessment. The framework encompasses both targeted and non-targeted analytical approaches, target selection strategies based on toxicological relevance and exposure prevalence, and the fundamental challenges posed by the vast chemical diversity of environmental contaminants spanning multiple orders of magnitude in physicochemical properties.

### 1.3. Scope and Objectives of This Narrative Review

This narrative review provides a comprehensive examination of multiclass assays for measuring environmental chemical mixture exposure, with particular focus on analytical methodologies that enable simultaneous quantification of diverse chemical classes in biological matrices [12,14,40]. This narrative review examines the development and application of multiclass assays for environmental chemical exposure measurement, focusing on analytical methodologies that enable simultaneous quantification of diverse chemical classes [41,42]. The scope encompasses mass spectrometry platforms, sample preparation strategies, and validation approaches for comprehensive exposure measurement in human biomonitoring studies, with particular emphasis on the technical challenges and solutions for analyzing complex chemical mixtures in biological matrices [40,41,43]. This review highlights the necessity for thorough analytical methods that include all significant environmental chemical classes in biological matrices to facilitate accurate exposure and risk evaluation [41,42,44]. Table 1 provides a comprehensive overview of recent multiclass analytical studies for environmental chemical exposure measurement, summarizing study designs, analytical platforms, target analyte numbers, and primary research objectives across diverse human biomonitoring applications. The methodology facilitates, for the first time, the concurrent characterization of the food-derived metabolites, endogenous metabolome, medicines, environmental contaminants, home chemicals, and microbial derivatives, encompassing over 1000 metabolites in total [14]. The distinction between exposomics and metabolomics is critical for understanding the scope of multiclass analytical approaches. While metabolomics focuses on comprehensive measurement of endogenous metabolites and biological responses, exposomics specifically targets environmental chemical exposures and their biomarkers throughout the human lifespan. The novel exposomics methodology complements metabolomics techniques and is scalable to facilitate extensive exposome research. Through systematic evaluation of current methodologies and their applications, this review aims to guide future developments in exposome-scale analytical approaches and their implementation in large-scale epidemiological studies (Table 1) [12,14,41].

## 2. Fundamentals of Multiclass Assay Development

**Figure 5:**
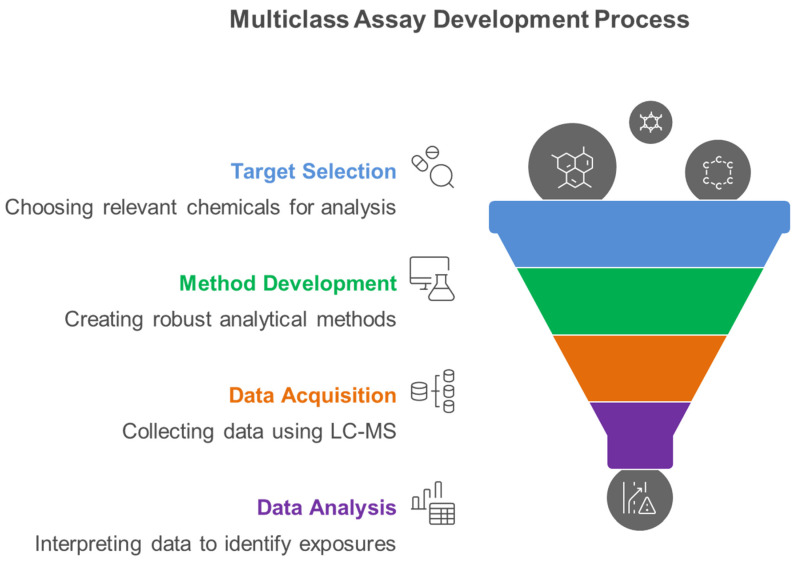
Multiclass Assay Development Process for Measuring Environmental Chemical Mixture Exposure.

Figure 5 illustrates the systematic four-phase workflow essential for developing robust multiclass analytical methods that can simultaneously quantify diverse environmental chemical classes, representing the methodological foundation for comprehensive exposome characterization in human biomonitoring studies. The systematic workflow encompasses four critical phases: target selection based on toxicological relevance and exposure prevalence, method development utilizing advanced LC-MS platforms, data acquisition through optimized analytical protocols, and comprehensive data analysis for exposome characterization.

### 2.1. Principles of Simultaneous Multi-Analyte Detection

The foundation of multiclass assay development lies in leveraging advanced mass spectrometry platforms that enable simultaneous detection and quantification of chemically diverse compounds exhibiting excellent sensitivity and specificity. Liquid chromatography coupled with mass spectrometry (LC-MS) is presently the leading technique in exposome-wide association studies (ExWAS) and is applicable for both targeted and non-targeted analysis [16,17,40]. Targeted assays concentrate on measuring a specific group of compounds with exceptional sensitivity, accuracy, and precision. In contrast, non-targeted analysis and suspect screening seek to identify and characterize chemicals without prior knowledge of the sample’s composition, potentially uncovering novel or unexpected exposures [24,40,42]. Owing to their excellent assay robustness, sensitivity, and quantitative capabilities, multi-analyte targeted LC-MS/MS approaches are frequently the preferred option, enabling the detection and quantification of a diverse array of compounds at trace concentration levels and forming the foundation for exposomics [16,23,24,45]. Multiple reaction monitoring is the preferred method for targeted analysis, relying on the observation of typically two transitions per analyte, which facilitates excellent sensitivity and specificity [45]. A triple quadruple mass spectrometer with an electrospray ionization (ESI) source was employed in the research, utilizing multiple-reaction monitoring (MRM) mode for data collecting of each target analyte [12,16]. The most significant primary ion transition was utilized for quantification, while the most intense secondary ion transition served for confirmation [12,16]. These instrumental capabilities form the technical backbone for achieving the sensitivity and selectivity required for comprehensive exposome analysis. Table 2 provides a comprehensive overview of mass spectrometry platforms, sample preparation strategies, chromatographic approaches, and validation parameters employed across multiclass environmental chemical analysis methods, demonstrating the diversity of analytical approaches used in exposome research.

### 2.2. Chemical Diversity Challenges in Environmental Exposomics

The simultaneous analysis of multiple chemical classes presents unprecedented analytical challenges due to the vast physicochemical diversity of environmental contaminants, spanning orders of magnitude in polarity, molecular weight, and chemical properties [16,22,46,47]. The wide range of structural and physicochemical characteristics of toxicants presents a significant challenge for the simultaneous analysis of various chemical classes [16]. The diverse chemical properties of multiclass CECs make their simultaneous analysis quite challenging. The extensive variety in physical and chemical properties, including the broad polarity range, poses a significant challenge for concurrent screening through a single LC-MS analysis. The varied chemical properties of the extracted compounds necessitate chromatographic columns capable of retaining molecules across a wide spectrum of polarities, while also efficiently separating multiple isomers that share the same mass to charge (*m*/*z*) ratios [16]. The extensive physicochemical variety of xenobiotics suggests a broad spectrum of toxicological impacts on humans, including liver carcinogenicity, nephrotoxicity, and estrogenicity (Figure 2) [22]. Approximately 5000 environmental chemicals are believed to be distributed and accumulated in humans. Residues can undergo various transformations into multiple metabolite and product forms through phase-I and -II reactions, including oxidation, hydroxylation, and dealkylation in biological metabolism, thereby broadening their characteristics and the list for screening exogenous mixtures. The integration and thorough assessment of the metabolome and exposome present significant challenges due to the vast range of concentrations of metabolites, drugs, food components, and environmental pollutants, which cover around ten orders of magnitude and include a wide variety of chemical classes [22]. These chemical diversity challenges necessitate sophisticated analytical strategies that can accommodate the full spectrum of environmental contaminants while maintaining analytical performance (Figure 4) [46,48]. The boundaries between exposome and metabolome research domains are defined by their distinct analytical targets and biological significance, though both utilize similar mass spectrometry platforms for chemical characterization. The exposome focuses on external environmental chemical exposures and their biomarkers, while the metabolome encompasses endogenous biochemical processes. The overlap occurs in the analytical detection of chemical compounds in biological matrices, where environmental chemicals typically detected at minimal levels (pg-ng/mL range) coexist with endogenous metabolites found at much higher concentrations (picomolar to millimolar range) [22,39]. This concentration gap of approximately 1000-fold between endogenous substances and environmental pollutants represents a key analytical challenge requiring specialized sample preparation and detection methods [28,35].

### 2.3. Target Selection Strategies for Comprehensive Coverage

Strategic selection of target analytes requires balancing toxicological relevance, exposure prevalence, analytical feasibility, and representativeness across chemical classes to ensure comprehensive exposome coverage [49,50,51]. The selection of chemicals was informed by their toxicological significance (i.e., is the compound relevant in a health context?), actual exposure levels (i.e., does this compound exist in quantities that can be measured?), compatibility with the instrumental platform (i.e., is retention through reversed-phase LC and ionization via ESI-MS adequate for sensitive analysis?), and being representative for each class of toxicants to ensure comprehensive monitoring of various adverse exposures [49,50]. Target analytes suitable for the multiclass HBM method were chosen according to priority lists of the U.S. Environmental Protection Agency (EPA), EDC-relevant EU legislations on endocrine disruptors, or novel or emerging compounds recently identified in the scientific literature [49,50,52]. The analyte panel was developed from prior research and covered air pollutants, disinfectants and their byproducts, endogenous estrogens, flame retardants, food processing by-products, industrial products, medical drugs, mycotoxins, personal care products, pesticides, phytoestrogens, phytotoxins, plastic-related chemicals, and PFAS compounds [11,50,53,54]. A multiclass targeted analyte list was established beforehand to direct method development, with the choice of anthropogenic contaminants, natural dietary substances, and tobacco markers limited to substances that are regularly tracked in human serum or urine by the US National Health and Nutrition Examination Survey (NHANES) and European HBM4EU priority substances [41,49,50]. The choice of target analytes and analyte classes aimed to be as extensive as technically possible, encompassing various structures, everyday life exposure levels, and toxicological pathways of action [41,51,54]. These systematic selection criteria ensure that multiclass methods capture the most toxicologically relevant and prevalent exposures while maintaining analytical feasibility for large-scale studies (Figure 4) [49,50,51].

## 3. Mass Spectrometry Platforms for Multiclass Analysis

### 3.1. Triple Quadrupole Mass Spectrometry (QQQ-MS/MS)

Triple quadrupole mass spectrometry represents the gold standard for targeted multiclass analysis, providing exceptional sensitivity and quantitative precision essential for trace-level environmental contaminant detection in biological matrices. A triple quadruple MS instrument featuring an Agilent Jet Stream electrospray ionization (AJS-ESI) source was utilized in studies, employing mass spectrometry in multiple-reaction monitoring mode (MRM) for data acquisition of each target analyte [39]. A Sciex 6500+ triple quadrupole mass spectrometer (MS) (SCIEX, Framingham, Massachusetts, USA) equipped with an electrospray ionization (ESI) source was employed in either positive or negative ionization mode, operating sequentially or concurrently, to detect and quantify the analytes of interest [12]. Although high-resolution, nontargeted methods are suggested as the future cornerstone for exposomics research, targeted methods using triple quadrupole instrumentation continue to deliver superior sensitivity, linearity, and less demanding data processing requirements [16,40,41]. Detection and quantification of biomarkers were carried out using a triple quadrupole mass spectrometer functioning in multiple reaction monitoring mode, utilizing both positive and negative electrospray ionization [29]. The superior quantitative capabilities of triple quadrupole systems make them indispensable for comprehensive exposome studies requiring precise measurement of hundreds of analytes.

#### 3.1.1. Multiple Reaction Monitoring (MRM) Optimization

Optimization of multiple reaction monitoring parameters constitutes a pivotal phase in methodological advancement, requiring systematic tuning of mass spectrometric conditions to achieve maximum sensitivity and selectivity for each target analyte. Enhancing the method for multiple reaction monitoring (MRM) transitions involved selecting precursor ion (Q1) and product ion (Q3), fine-tuning entrance potential (EP), collision energy (CE), and collision cell exit potential (CXP) [24]. Values of Q1, Q3, EP, and CE were refined for numerous compounds on a new Qtrap 7500 instrument, utilizing method parameters from previous methods on a Qtrap 6500+ as the foundation [24]. Parameters specific to the analyte, including declustering potential, entrance potential, collision exit potential, and collision energy, were optimized individually through direct syringe infusion of each compound into the mass spectrometer [12]. Two fragment ions were selected for each analyte as quantifier and qualifier ions. To ensure optimal data quality, dwell periods were modified for all analytes to secure adequate data points for each chromatographic peak [24]. The MRM method utilizing quantifier and qualifier ion transitions enables both detection and quantification, as well as identification and confirmation, which is crucial for targeted analyses [12]. These comprehensive optimization procedures ensure robust analytical performance across diverse chemical classes while maintaining the specificity required for complex mixture analysis (Table 2) (Figure 5).

#### 3.1.2. Sensitivity and Selectivity Considerations

The exceptional sensitivity achieved through optimized triple quadrupole methods enables detection of environmental chemicals at extremely low concentrations, with limits of detection typically in the sub-ng/mL range across diverse biological matrices [11,55,56,57,58,59]. For the majority of compounds, the detection and quantitation limits (LODs/LOQs) were found to be within the range of 0.001–0.1 ng/mL [16]. Detection limits varied from 0.01 to 1.0 ng/mL of urine, with most being ≤0.5 ng/mL (42 out of 50) [12]. The evaluation of method robustness showed appropriate extraction recovery and matrix effects (SSE) ranging from 60 to 130%, inter-/intra-day precision (RSD) below 30%, and outstanding sensitivity (limit of detection, 0.015–50 pg/mL) for 60–80% of the analytes across the studied matrices [24]. The method shows remarkable sensitivity with detection limits ranging from 0.01 to 1.0 ng/mL, outstanding precision with relative standard deviations under 20%, and extraction recoveries ranging from 80% to 110% [29]. These sensitivity achievements enable reliable quantification of environmental exposures at levels relevant for human health risk assessment [11,55,56,57,58]. Table 3 provides comprehensive detection performance data for multiclass analytical methods across diverse chemical classes and human biological matrices, demonstrating the analytical capabilities achieved in exposome research.

### 3.2. High-Resolution Mass Spectrometry (HRMS)

High-resolution mass spectrometry platforms offer complementary capabilities for multiclass analysis, providing accurate mass determination and structural elucidation essential for non-targeted screening and discovery of unknown exposures [41,48,51,60,61,62,63]. Liquid chromatography high-resolution mass spectrometry-based approaches offer the potential to enhance our understanding of the exposome in future exposome-wide association studies, as this technique can identify a wide range of small molecules with various chemical properties, from environmental xenobiotics to endogenous metabolites [41,51,60,62,64]. External stressors, such as xenobiotics and environmental changes, are assessed concurrently alongside phenotypical changes resulting from these exposures, positioning LC-HRMS as an excellent platform for creating comprehensive methods to investigate the exposome [41,51,60,62]. Advancements in high-resolution mass spectrometry (HRMS) have resulted in an increasing number of HRMS-based screening methods being published in recent years [41,48,51,60,61,65,66]. While these methods could cover a broader chemical space, they presently lack the necessary sensitivity to effectively monitor a large number of pollutants in the environment at ultra-trace levels [41,60,64]. The broad chemical coverage and retrospective analysis capabilities of HRMS make it invaluable for comprehensive exposome characterization [41,51,61,62,67].

#### 3.2.1. Data-Dependent and Data-Independent Acquisition Approaches

Data-dependent or independent acquisition strategies enable systematic fragmentation of detected compounds, providing structural information essential for compound identification and confirmation in complex biological matrices [68,69]. Data-dependent acquisition (DDA) focuses on choosing the n “*n*” most intense *m*/*z* detected at the MS1 level for subsequent selection and fragmentation, typically resulting in high-quality MS2 spectra of several detected features [25]. Data-dependent acquisition (DIA) focuses on the systematic separation and fragmentation of all precursor ions across an extensive window, facilitating a thorough examination of mass spectra [25]. Data-dependent acquisition utilizing an inclusion list of the precursor ions was employed for analyte confirmations [35]. Data-dependent acquisition was used for analyte confirmation, as it provides higher confidence and cleaner MS2 spectra [34]. Since the levels of exogenous residues in serum are typically 100–1000 times lower than those of internal metabolites, it is challenging to acquire adequate fragments of external residues through multiple DDA during a single injection screening [37]. Despite the challenges related to concentration, the DDA and DIA strategies offer crucial structural information that enables confident compound identification, provided they are properly optimized. This is especially true when these strategies are used in conjunction with accurate mass determination and spectral library matching techniques in non-targeted exposome studies [68,69].

#### 3.2.2. Accurate Mass Determination and Structural Elucidation

Accurate mass determination combined with MS/MS fragmentation patterns enables confident structural elucidation and compound identification, supporting both targeted quantification and discovery of unknown exposures [48,70,71,72,73,74]. To ensure the precision of MS1 and MS2 data, the precursor ions and fragments from clean standards produced experimentally must align with theoretic calculations within ±10 ppm [37]. In MS1 searching, the variations in retention time and mass accuracy across experimental data and the database were established from ±0.30 min and ±10 ppm, respectively. Compounds that achieved an MS1 score above 50 were categorized as tentatively positive findings. [37]. Following the identification of potential candidates whose precursors exhibited mass differences aligned with the suspect list (Δmass < 5 ppm), a comparison was made between their MS/MS spectra and those found in the Mass Bank database or predicted through MetFrag [38]. Spectral matching for annotations with a confidence level of 2 takes into account an accurate MS1 mass deviation of up to 0.005 Da within the detected precursor ion and the library record, along with a total identification score exceeding 700 [34]. These structural elucidation capabilities enable discovery and identification of previously unknown exposures in human biomonitoring studies [48,62,70,71,72,73,74,75]. Computational tools and databases play a critical role in non-targeted identification of environmental chemicals in exposome research. Once potential candidates with precursors mass matched to suspect lists are identified, MS/MS spectra are compared with those located in the MassBank database or predicted via MetFrag for structural elucidation [38]. Spectral matching for annotations considers accurate MS1 mass deviation and total identification scores, with European Mass Bank, MS-DIAL, and Mass Bank of North America databases being used for metabolite annotation [34,35]. These computational approaches enable confident identification of previously unknown exposures while maintaining appropriate confidence levels for structural assignments.

### 3.3. Hybrid Approaches and Method Integration

Integration of targeted and non-targeted approaches within single analytical workflows maximizes the benefits of both methodologies, enabling sensitive quantification of priority analytes while maintaining discovery potential for unknown exposures [76]. A single-injection LC-MS approach to integrated exposomics and metabolomics, whether targeted or untargeted, merges the strengths of both strategies, offering high sensitivity through targeted analysis and extensive coverage with the ability for retrospective data examination in untargeted analysis [25]. The implementation of Zeno technology, a linear ion trap stage preceding the TOF analyzer that enables duty cycles of up to 90%, leads to notable enhancements in sensitivity [25]. The results showed a notable enhancement in sensitivity, as evidenced by the ability to detect lower concentrations in spiked SRMs. The mean values for SRMs 1950 [77] and 1958 [78] were 2.2 and 3 times lower, respectively. Additionally, there was an overall increase in detection frequency by sixty-eight percent in the MRM-HR + SWATH mode as opposed to the SWATH-only approach [25]. The integrated approach of targeted and untargeted chemical exposomics facilitated accurate and detailed measurement of key analytes while also allowing for the identification of unforeseen exposures that may have health implications. These hybrid approaches represent the future of exposome analysis, combining the quantitative precision of targeted methods with the discovery potential of non-targeted screening in a single analytical workflow.

## 4. Sample Preparation Strategies for Diverse Chemical Classes

### 4.1. Extraction Method Development and Optimization

Effective sample preparation represents the cornerstone of successful multiclass analysis, requiring sophisticated extraction strategies that can simultaneously recover chemically diverse compounds while minimizing matrix interferences and maintaining analytical performance [17,23,79,80,81]. The creation of multiclass assays necessitates careful refinement of the complete analytical workflow, influencing sample preparation procedures, to minimize interferences, isolate, and concentrate analytes across various matrices [23]. Effective sample pretreatment prior to instrumental analysis can minimize interferences, isolate, and enhance analytes in different matrices, and numerous sample preparation methods have been refined to examine the exposome [17,23,79]. The presence of target analytes with diverse physicochemical properties, such as polarity, poses an important hurdle for solid-phase extraction techniques in exposomics, stemming from the differences in analyte-sorbent interactions [17,23,80]. Appropriate sample preparation methods are crucial, as components of the sample matrix can significantly influence signal intensities, especially for trace level compounds. Additionally, a limited duty cycle can affect data quality in extensive LC-MS/MS assays aimed at analyzing hundreds of analytes in one run [23,79,80]. The selection and optimization of extraction methods must balance chemical coverage, recovery efficiency, and practical considerations for high-throughput applications [17,23,80,81,82].

#### 4.1.1. Liquid–Liquid Extraction Approaches

Liquid–liquid extraction methods offer broad chemical coverage and compatibility with diverse analyte classes [83,84], making them particularly suitable for comprehensive exposome analysis despite requiring careful optimization to achieve acceptable recoveries across all target compounds. The sample preparation involved two rounds of liquid–liquid extraction using 3 mL of a combination of ethyl acetate and n-hexane (3:2, *v*/*v*) with 0.6% formic acid. Prior to the extraction procedure, 200 μL of plasma was combined with isotope-labeled surrogate standards to compensate for analyte loss throughout sample preparation and to mitigate any matrix effects [28]. The suggested LLE method aligns better with a variety of target CEC classes compared to solid-phase extraction using different sorbents, and conventional SPE-based methods tend to be more time-intensive and significantly pricier than the LLE approach [28]. For serum and urine extraction, a volume of 200 μL was augmented with 10 μL of internal standard solution and extracted using 790 μL of ACN/MeOH (1/1) via sonication (10 min, 4 °C), followed by protein precipitation during a freeze-out phase (2 h, −20 °C) [11]. The liquid extraction and precipitation of proteins are commonly employed extraction methods in exposomics because of their relatively broad chemical coverage [23]. These liquid–liquid extraction approaches provide cost-effective and broadly applicable methods for multiclass compound recovery [84], though they may require additional cleanup steps for optimal performance.

#### 4.1.2. Solid-Phase Extraction Techniques

Solid-phase extraction (SPE) represents the preferred approach for many multiclass applications, offering superior cleanup capabilities, enhanced selectivity, and improved method robustness compared to traditional liquid–liquid extraction methods [85,86]. SPE is typically recognized for its effective cleanup, enhanced selectivity, and reduced solvent consumption, making it appropriate for large sample sizes and high throughput, especially when compared with liquid–liquid extraction (LLE) in a multi-class method [12]. Oasis HLB SPE, a polymeric sorbent with a balanced hydrophilic-lipophilic nature, was found to be appropriate for this study after evaluating different sorbent materials; it has also been a favored universal sorbent in various multi-class methods [12]. A refined SPE protocol employing 96-well plates was implemented based on a recently established method, in which the SPE was preconditioned with methanol (MeOH) and subsequently with water (H2O) [24]. Recently, solid-phase extraction has garnered increased attention as an effective method for sample preparation in exposomics and is considered a promising strategy for future large-scale applications due to its ability to mitigate matrix effects, improve sensitivity, and ensure consistency in high-throughput studies [23]. The hydrophilic-lipophilic-balanced sorbent made from the polymeric N-vinylpyrrolidone-divinylbenzene is a flexible sorbent known for its effective recovery in extracting substances from water and wastewater. A 96-well plate version of HLB-SPE has been previously utilized for blood extraction [19]. The versatility and automation potential of SPE methods make them particularly attractive for large-scale exposome studies requiring consistent performance across thousands of samples [23,86].

#### 4.1.3. Passive Equilibrium Sampling Methods

Passive equilibrium sampling (PES) offers a unique approach for extracting neutral and hydrophobic compounds from biological matrices, providing clean extracts with minimal matrix interference while being particularly effective for lipophilic environmental contaminants [87,88,89]. Passive equilibrium sampling is an approach developed for extracting compounds from tissue and biological samples, involving the placement of a defined mass of polymer into the sample, allowing chemicals to be extracted through diffusion directly into the polymer [19]. The selected polymer was polydimethylsiloxane, known for its ability to extract a range of hydrophobic as well as neutral chemicals with fairly rapid uptake kinetics, attributed to the high diffusion constants within the PDMS, though it can only extract charged organic molecules to a very limited extent [19]. PES using PDMS provides clean extraction of hydrophobic and neutral compounds without co-extracting undesirable matrix components such as proteins and salts, and the minimal lipid uptake that may occur from lipid-rich matrices does not compromise analytical performance in plasma samples [19]. While PES methods provide excellent cleanup for hydrophobic compounds, their limited applicability to polar and ionic species necessitates combination with complementary extraction techniques for comprehensive coverage [90].

### 4.2. Matrix-Specific Considerations

#### 4.2.1. Biological Matrix Challenges

Biological matrices present complex analytical challenges due to high concentrations of endogenous compounds, varying protein and lipid content, and the need to extract trace-level contaminants from matrices dominated by endogenous substances [91,92]. Human biomonitoring samples can include various matrices, with typical examples being blood (full blood, serum, and plasma), breast milk, urine, and even organs like the placenta or post-mortem tissues such as the liver, brain, and adipose tissue [19]. The issue with chemical exposomics in plasma from humans lies in the significant concentration disparity, with endogenous substances being 1000 times more prevalent than environmental pollutants [34,93]. Phospholipids constitute the primary endogenous small molecules found in plasma, characterized by a rich and diverse mixture of more than 2000 chemical species [34]. Given the elevated fat and protein levels in milk, a two-phase extraction procedure was employed to reduce the organic layer of the matrix components [16]. The cerebral spinal fluid (CSF) is the most closely associated biological fluid with the brain, meaning that any deviations in the matrix are directly linked to pathological changes occurring in the brain [36]. Although it holds biological importance, the quantity of metabolomics/exposomics studies conducted on CSF samples is limited due to the invasive and valuable nature of the sample (which necessitates a lumbar puncture) along with methodological hurdles, such as the absence of standard materials and the relatively low concentrations of chemicals in CSF relative to other matrices like blood [36]. These biological matrix challenges require specialized extraction and cleanup procedures to achieve adequate sensitivity for trace-level environmental contaminants while managing high concentrations of interfering endogenous compounds [17,91].

#### 4.2.2. Environmental Sample Complexity

Environmental samples present additional complexity due to variable matrix composition, potential for high levels of interfering compounds, and the need to accommodate samples with widely varying chemical compositions and concentrations [91,94,95]. The enhanced “all-in-one” method combined with QQQ MS was utilized to assess the concentrations of CECs in a range of aqueous specimens, which include two human urine samples, three sewage effluents, and thirteen surface waters [39]. The matrix effects from urine were more pronounced than those from the sewage effluent, probably because of the increased levels of urea, inorganic salts, and creatinine present in urine matrices [39]. The salt constituents in sample matrices might reduce the trapping efficiency and present significant matrix effects on the CECs that eluted on the MMIE column [39]. Environmental sample complexity necessitates robust analytical methods capable of handling variable matrix compositions while maintaining consistent analytical performance [91,95,96].

### 4.3. Recovery and Matrix Effect Assessment

Comprehensive evaluation of extraction recovery and matrix effects across all target analytes is essential for method validation and ensuring reliable quantitative results in multiclass exposome analysis [12,14,16,19,62]. The extraction efficiency from urine and serum samples were elevated (median of 93% and 87%, respectively), whereas recoveries from breast milk were diminished (median of 54%) [16]. Extraction recoveries ranged from 83 to 109% [12]. Matrix effects were mitigated through the utilization of matrix-matched calibration standard curves and stable isotope-labeled internal standards [12]. Signal suppression or enhancement (SSE) was assessed as the ratio of the slope of the calibration curve created in a urine matrix to that of a curve constructed in a reagent-based water [12]. The average chemical recovery were 61% for SolvPrec, 38% for PES + SPE, and 27% for SPE, with PES + SPE significantly improving the mean chemical recovery relative to SPE, particularly for neutral hydrophobic compounds [19]. The use of internal standards for recovery correction in bioassays is unfeasible and presents difficulties for analytical target lists containing numerous chemicals, necessitating the attainment of consistent and uniformly distributed recoveries across hydrophilic to hydrophobic, charged to neutral, and to nonpersistent to persistent compounds [19]. Systematic assessment of recovery and matrix effects ensures that multiclass methods provide reliable quantitative data across the full range of target analytes, forming the foundation for accurate exposome characterization and subsequent health risk assessment [11,14,62].

## 5. Chromatographic Separation Strategies

### 5.1. Reversed-Phase Liquid Chromatography

Reversed-phase (RP) liquid chromatography serves as the primary separation technique for multiclass environmental analysis, providing effective retention and separation for the majority of target compounds across diverse chemical classes [97,98,99]. A RP column (Poroshell 120 EC-C18, 3.0 mm × 50 mm, 2.7 μm) was used as the first column to enrich and separate the majority of the CECs [39]. As expected, a majority of the CECs (compounds **1**–**87**) demonstrated appropriate retention times and acceptable peak forms on the RP column [39]. However, a small subset of CECs (compounds **88**–**102**) showed minimal retention, eluting near the void time with poor chromatography peaks [39]. Chromatographic separation was achieved using a Zorbax Eclipse Plus RRHD C18 column (2.1 × 50 mm, 1.8 μm; with corresponding Eclipse Plus guard column; Agilent Technologies, Wilmington, Delaware, USA) and a Zorbax Eclipse Plus C18 RRHD delay column (4.6 × 50 mm, 3.5 μm) [31]. The analytical coverage is primarily influenced by the capabilities of various liquid chromatography configurations in capturing and separating molecules, with a one-dimensional reversed-phase C18 liquid chromatography concentrating on the analysis of exogenous chemical residues with medium and low polarity [37]. Specific liquid chromatography configurations utilizing phenyl columns and polar embedded columns were designed for the analysis of higher polarity residues and residue metabolites with enhanced polarity [37]. While reversed-phase chromatography provides excellent separation for most environmental contaminants, its limitations with highly polar and ionic compounds necessitate complementary separation strategies [98,100,101].

### 5.2. Mixed-Mode and Ion Exchange Chromatography

Mixed-mode and ion exchange (MMIE) chromatographic approaches address the limitations of traditional reversed-phase separation by providing retention mechanisms for highly polar and ionic compounds that are poorly retained on conventional C18 columns [102,103]. The MMIE column, examined as a third option, demonstrated the best performance among the three candidate columns [39]. This is likely due to its trimodal separation mechanisms, which include reversed phase, strong cation exchange, and weak anion exchange. It is compatible with aqueous samples and can separate both cationic and anionic compounds, thereby minimizing the effect of the residual aqueous solvent on the peak shapes [39]. A homemade trap column (3.0 mm × 20 mm) filled with a layered combination of WAX and WCX sorbents (Strata X-AW, Strata X-CW, Phenomenex Inc., Torrance, California, USA) was employed to trap ionic and highly polar CECs that are not retained by the RP column [39]. The mixed-mode sorbent was synthesized by combining equal weights of two solid-phase extraction sorbents, main secondary amine (PSA) and C18 (PSA + C18), prior to packing [23]. Given the robust anion-exchange interactions of PSA with frequently encountered acidic chemicals in exposomics, a basic methanol solution (3% NH3) was employed as the elution solvent to evaluate the recovery of the mixed-mode sorbent [23]. These mixed-mode approaches significantly expand the analytical coverage of multiclass methods by accommodating compounds across the full polarity spectrum [102].

### 5.3. Column-Switching and Multi-Dimensional Approaches

Column-switching and multi-dimensional chromatographic systems enable comprehensive analysis of chemically diverse compounds by combining multiple separation mechanisms within a single analytical run, maximizing both coverage and efficiency [39,104,105,106,107,108]. The concept of an “all-in-one” LC-MS system, or a “column-switching method”, employed in previous studies for the analysis of all chain-length PFAS, has been adapted with modifications in this study [39]. Instead of using two duo two-position, eight-port 2D-LC valves, two switching valves were implemented: a two-position, six-port valve and a two-position, 10-port valve, which are more commonly found in LC systems [39]. When the trap column was connected to the RP column, the highly polar and ionic CECs were enriched and trapped in the trap column during the first 6.5 min of the run [39]. Then, the valves were switched to position II, placing the trap column in standby mode, while the majority of CECs (compounds **1**–**87**) were separated on the RP column and analyzed by MS [39]. A 2D-LC separation system was modified using an Agilent UPLC combination system, where a short Acquity BEH C18 column (2.1 × 5 mm, 1.7 μm) served as the pre-column to separate the initial sample into two fractions: a more polar portion and a less polar/nonpolar fraction [37]. A Discovery HS F5-5 column (2.1 × 50 mm, 3.0 μm) was optimized for the separation of relatively polar compounds, and for the segregation of less polar and nonpolar molecules, an Acquity BEH C18 column (2.1 × 50 mm, 1.7 μm) was used [37]. The methodology employs a dual-column system, utilizing both a reversed-phase (RP) column and a hydrophilic interaction liquid chromatography (HILIC) column operating concurrently to address polar chemicals and predominantly nonpolar xenobiotics [22,60]. These multi-dimensional approaches represent a significant advancement in analytical capability, enabling comprehensive coverage of the chemical exposome within practical analysis times [41,106,107,108].

### 5.4. Large Volume Injection Techniques

Large volume injection techniques provide a powerful approach for enhancing method sensitivity by concentrating analytes at the column head, enabling detection of trace-level contaminants without extensive sample preconcentration steps [39,109,110,111]. Large volume injection (LVI) is another technique that can enhance method sensitivity via direct injection of a large sample volume into a LC column with minimal pretreatment [39]. The target analytes are concentrated at the column head and subsequently separated by gradient elution [39]. For the setup of LVI (900 μL), the RP gradient started at 97% of the aqueous mobile phase at a flow rate of 0.6 mL min^−1^, held for 6.5 min, with the injection valve in the main pass position [39]. LVI significantly increased the method sensitivity, achieving quantifying limits for 93 of the 102 CECs in different water matrices at <10 ng L^−1^ [39]. However, LVI also faces several limitations, as analytes with poor retention on LC columns exhibit deteriorated peak shapes, and matrix effects pose great challenges, particularly for the analytes that elute early, which compromises the quantitative accuracy and necessitates that the initial column eluate be discarded [39]. Greater injection volume with little matrix interference enabled precise multiclass targeted detection of 77 key analytes: Median MLOQ = 0.05 ng/mL for 200 μL of plasma [34]. While large volume injection techniques offer significant sensitivity enhancements [39,110,111,112], their implementation requires careful consideration of matrix effects and chromatographic performance to maintain analytical quality across all target analytes [39,109].

## 6. Biological Matrix Applications

**Figure 6:**
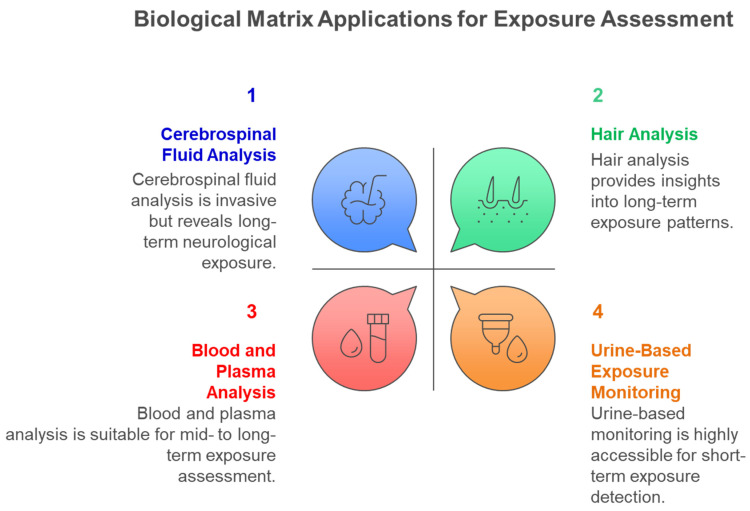
Biological Matrix Applications for Multiclass Environmental Chemical Exposure Assessment.

**Figure 7:**
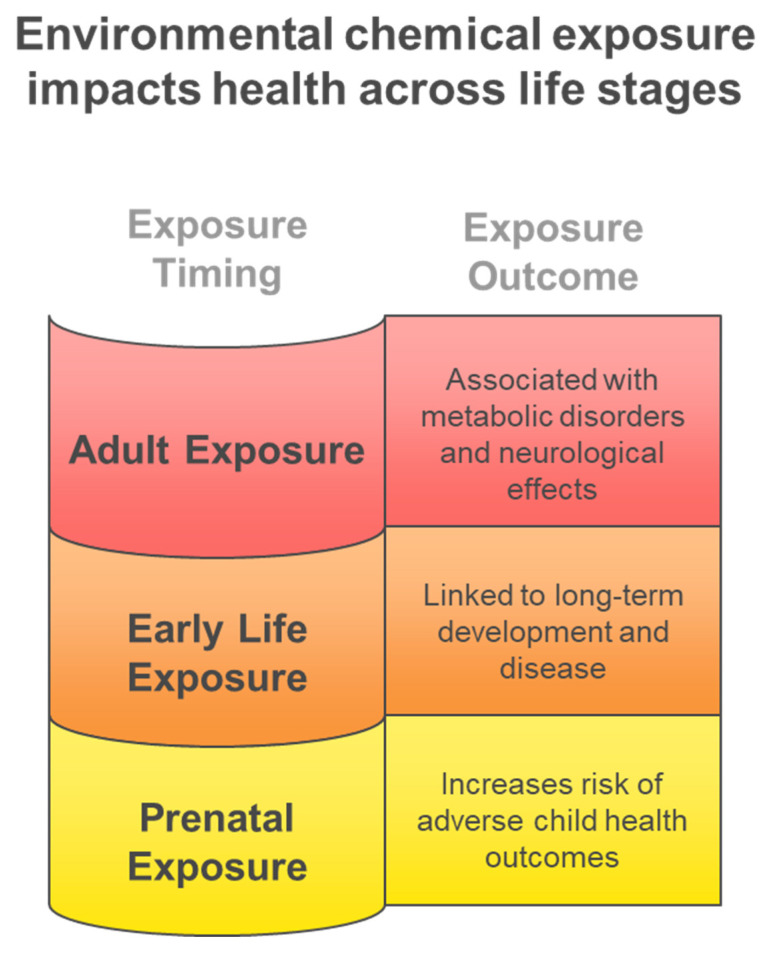
Environmental chemical exposure impacts health across life stages.

Figure 6 illustrates the diverse biological matrices employed in multiclass exposome analysis, each providing unique temporal windows and accessibility characteristics that complement comprehensive environmental chemical exposure assessment across different study designs and population requirements. Cerebrospinal fluid provides direct evidence of central nervous system exposure despite its invasive collection requirements, hair analysis enables assessment of chronic exposure patterns over extended periods, blood and plasma analysis offers systemic exposure characterization with moderate temporal resolution, and urine-based monitoring provides highly accessible short-term exposure detection capabilities for large-scale biomonitoring studies. Figure 7 illustrates the critical importance of exposure timing in determining health outcomes, demonstrating how the exposome paradigm encompasses diverse environmental influences across the entire lifespan, from prenatal development through adulthood, each presenting unique vulnerabilities and health consequences. Early life exposures are particularly critical for long-term health outcomes, while adult exposures contribute to chronic disease development.

### 6.1. Blood and Plasma Analysis

#### 6.1.1. Comprehensive Plasma Exposome Characterization

Comprehensive plasma exposome characterization enables simultaneous quantification of diverse chemical classes in blood samples, revealing the complex mixture of environmental contaminants present in human circulation [15,18,34,113,114,115]. Plasma samples were produced and analyzed using a previously established methodology combining untargeted and targeted chemical exposomics, with the chemical exposomics method being validated for 83 targeted analytes, including dietary chemicals, environmental contaminants, drugs, tobacco markers, and endogenous steroid hormones [35]. Among all plasma samples, 57 of the 83 target analytes were detected and quantified, with these substances belonging to 14 diverse chemical classes, including pesticides (organophosphate and neonicotinoid), flame retardants, PFAS, personal care products, pharmaceuticals, plasticizers, dietary substances, polycyclic aromatic compounds, a nicotine metabolite, and endogenous steroid hormones [35]. In each adult plasma (100 μL, n = 34), 28 analytes were identified and quantified across 10 chemical classes, with the quantification of per- and polyfluoroalkyl compounds externally verified using independent targeted analysis [34]. The methodologies have been effectively verified for the analysis of human blood concerning manmade phenolic compounds, with 21 analytes assessed in Swedish young adults, revealing the presence of synthetic phenolic antioxidants in numerous persons at elevated blood concentrations [20]. These comprehensive characterization studies demonstrate the feasibility of detecting complex chemical mixtures in plasma samples, providing a foundation for understanding systemic exposure patterns (Table 3) [15,34,114]. Recent applications of multiclass exposome methods have demonstrated direct quantitative relationships between chemical exposures and health outcomes through sophisticated statistical modeling approaches. Studies utilizing Bayesian kernel machine regression analysis have revealed significant associations between co-exposure to chemical mixtures and disease risk, with joint effects being statistically significant across exposure percentiles [28]. For instance, mixed-effect modeling has identified statistically significant exposome-metabolome interactions indicative of endocrine disruption, with positive correlations observed between testosterone and several PFAS compounds remaining significant in adjusted models [35]. Additionally, multiclass methods have enabled identification of significant positive associations between oxidative stress biomarkers and urinary biomarkers of exposure to various xenobiotics including flame retardants, pesticides, phthalates, and volatile organic compounds [29].

#### 6.1.2. Longitudinal Exposure Assessment

Longitudinal exposure assessment in plasma samples reveals the temporal dynamics of chemical exposures and enables identification of stable versus variable exposure patterns over time, providing critical insights for exposure misclassification in epidemiological studies [116,117,118]. This study applied chemical exposomics to recurrent plasma samples of 46 healthy Swedish adults, each sampled 6 times over 2 years in a multiomics wellness profiling study [35]. Repeated sampling of identical individuals over time facilitated the identification of novel exposure types and uncovered both unusual and common co-exposures pertinent to precision health [35]. The majority of annotated chemicals in plasma (306 of 519 analytes) exhibited ICCs < 0.40, with the mean ICC substantially greater for endogenous metabolites (0.40) compared to the chemical exposome (0.30, Student’s *t* test, two-tailed, *p* < 0.001) [35]. The PCA scores plot, differentiated by S3WP participant ID, frequently clustered the six samples from each subject, suggesting a consistent stability of the target exposome throughout a two-year period [35]. These longitudinal studies underscore the significance of repeated assessments of the chemical exposome in epidemiological research to reduce exposure misclassification. In longitudinal multiomics studies, the exposome should be evaluated as frequently, or more frequently, than other biomolecular profiles [116,117,118].

### 6.2. Urine-Based Exposure Monitoring

#### 6.2.1. Pediatric Population Studies

Pediatric population studies using urine-based exposure monitoring reveal unique exposure patterns in children and highlight the vulnerability of early life stages to environmental chemical exposures [32,119,120,121]. This research utilized the nationwide Environmental Impacts on Child Health Outcomes (ECHO) Cohort to evaluate chemical exposures in 201 children aged 2 to 4 years from 2010 to 2021 [32]. A total of 111 analytes from various chemical classes were concurrently measured in individual spot urinary specimens obtained from all children and their mother during pregnancy, with concentrations assessed between kid and prenatal maternal samples [32]. Of the 111 chemicals, 96 were identified in a minimum of five children, whereas 48 analytes were present in more than 50% of the children [32]. Thirty-four substances were universally identified (>90%), nine of which are absent from the United States national biomonitoring: triethyl phosphate, benzophenone-1, and six phthalate metabolites together with one alternative plasticizer [32]. After having demonstrated the feasibility of urine collection at this young age group, NHANES recently expanded its protocols to include the collection of urine specimens from children aged 3–5 years, beginning with the 2015–2016 survey period, for the measurement of nonpersistent chemical biomarkers [32]. These pediatric studies demonstrate widespread exposure to environmental chemicals in young children [32,119,121] and identify emerging contaminants not previously monitored in national surveillance programs [32].

#### 6.2.2. Adult Exposure Patterns

Adult exposure patterns revealed through comprehensive urine analysis demonstrate the complexity of environmental chemical exposures in the general population and provide insights into sources and determinants of exposure variability [122,123,124,125,126]. The assay was validated to quantify 50 exposure biomarkers in urine, categorized into 7 chemical classes and 16 sub-classes, encompassing metabolites of 5 polycyclic aromatic hydrocarbons (PAHs), 12 personal care and consumer product chemicals (PCPs), 18 pesticides, 5 organophosphate flame retardants (OPFRs), 4 tobacco alkaloids, 5 volatile organic compounds (VOCs), and 1 drug of abuse [12]. Human urine (0.2 mL) was augmented with isotope-labeled internal standards, enzymatically deconjugated, subjected to solid-phase extraction, and evaluated via high-performance liquid chromatography-tandem mass spectrometry [12]. A multi-class analytical technique for measuring biomarkers of environmental chemical exposure and biological responses in human urine has been created and validated, facilitating the simultaneous study of 125 biomarkers spanning 10 chemical classes, consisting of parent compounds and/or metabolites of volatile organic compounds, tobacco smoke, psychosocial stress, polycyclic aromatic hydrocarbons, phytoestrogens, phthalates and phthalate alternatives, pesticides, personal care and consumer product chemicals, oxidative stress, and flame retardants [29]. These adult exposure studies reveal significant inter-individual variability in chemical exposures and highlight age-related differences in exposure patterns and metabolic capacity [122,123,124].

### 6.3. Alternative Matrices for Exposure Assessment

#### 6.3.1. Hair Analysis for Long-Term Exposure

Hair analysis provides a unique window into long-term exposure patterns, offering integrated exposure assessment over extended periods and revealing chronic exposure patterns not captured by traditional biomonitoring approaches [127,128,129]. A study assessed the correlation between multiple classes organic contaminants and sex steroid hormones using hair analysis in 196 healthy Chinese women between 25 and 45 years [33]. Hair analysis has been employed to investigate the impact of a combination of 19 pesticides from various chemical classes and a mix of 13 PAHs on hormones levels in female rats, revealing significant reductions in hair E2 concentration due to pesticide exposure and in hair thyroid hormone concentrations due to PAH exposure [33]. Hair measurements indicate average analyte levels over prolonged durations, potentially spanning several months, contingent upon the length of hair sections analyzed; hence, short-term exposure fluctuations exert no impact on the amounts identified in hair [33]. The outcomes of this study revealed that each hormone correlated with a combination of at least 10 analyzed pollutants, with hair E2 content linked to 19 contaminants, including propoxur, γ-hexachlorocyclohexane, fipronil, permethrin, prochloraz, mecoprop, and carbendazim [33]. Hair analysis demonstrates the potential for assessing chronic exposure patterns and their associations with endocrine disruption, providing insights into long-term exposure-health relationships (Figure 6) [33,128,130,131].

#### 6.3.2. Cerebrospinal Fluid for Neurological Exposure

Cerebrospinal fluid analysis represents a specialized application for assessing chemical exposure to the central nervous system, providing direct evidence of blood–brain barrier penetration and potential neurological effects of environmental contaminants [132,133]. Cerebrospinal fluid is the most proximate biological fluid to the brain, with anomalies in this matrix directly correlating to pathological alterations in the brain (Figure 6) [36]. Notwithstanding its biological importance, the quantity of metabolomics/exposomics studies conducted on cerebrospinal fluid (CSF) samples is limited due to the invasive and valuable nature of the sample (necessitating lumbar puncture) coupled with methodological obstacles, such as the absence of standardized materials and the fairly low levels of chemicals in CSF relative to other matrices like blood [36]. The 28 pollutants analyzed displayed median values varying from the LOD to 10.5 ng/mL in blood and from LOD to 1.2 ng/mL in CSF. Four PFAS compounds were found in at least 60% of cerebrospinal fluid samples (n ≥ 108), namely perfluorobutanesulfonic acid (PFBS), perfluorooctanoic acid (PFOA), 6:2 chlorinated polyfluoroalkyl ether sulfonate (6:2 Cl-PFESA), and perfluorohexanesulfonic acid (PFHxS), with median concentrations varying from 0.001 to 0.042 ng/mL [36]. Research indicates that several environmental pollutants can penetrate the blood- brain barrier into the brain’s central nervous system (CNS), thereby impairing neuronal development and functions (Figure 2) [36]. CSF analysis provides critical insights into central nervous system exposure to environmental chemicals and demonstrates that molecular size is a key determinant of blood–brain barrier penetration, with important implications for neurotoxicity assessment [132,133].

## 7. Method Validation and Quality Assurance

### 7.1. Analytical Performance Criteria

#### 7.1.1. Sensitivity and Detection Limits

Achievement of exceptional sensitivity with detection limits in the sub-ng/mL range represents a critical requirement for multiclass exposome methods, enabling quantification of environmental contaminants at levels relevant for human health risk assessment [12,14,17,23,62]. For the majority of chemicals, the limits of detection and quantitation (LODs/LOQs) were within the range of 0.001–0.1 ng/mL [16]. The detection limits varied between 0.01 and 1.0 ng/mL in urine, with most of them being ≤0.5 ng/mL (42 out of 50) [12]. LVI significantly improved technique sensitivity, achieving quantification limits below 10 ng L^−1^ for 93 out of 102 CECs across diverse water matrices [39]. The robustness of the method was assessed, showing adequate extraction recovery and matrix effects (SSE) between 60 and 130%, inter-/intra-day precision (RSD) under 30%, and remarkable sensitivity (limit of detection, 0.015–50 pg/mL) for 60–80% of the analytes throughout the examined matrices [24]. The detection limits for human estrogens and xenobiotics varied from 0.01 to 5.7 ng/mL, with a median of 0.08 ng/mL in the solvent. In the matrix, the median limit of detection rose to 0.7 ng/mL in urine and 0.5 ng/mL in plasma, possibly attributable to matrix effects and interferences [22]. The technique detection limit ranged from 0.003 to 0.838 ng/mL, while the limit of quantification (LOQ) spanned 0.009 to 2.794 ng/mL. The accuracy of all substances was from 85% and 115%, while the precision was below 15% [13]. The overall technique sensitivity was rated as excellent to satisfactory for the majority of targeted analytes, with a median MLOQ of 0.05 ng/mL. Specifically, 62% of analytes exhibited MLOQ values ranging from 0.01 to 0.1 ng/mL, 34% from 0.2 to 1 ng/mL, and a mere 4% from 1 to 5 ng/mL [34]. These exceptional sensitivity achievements enable detection of environmental exposures at concentrations that are toxicologically relevant and support comprehensive characterization of the human exposome (Table 3) [12,14,17,23,62].

#### 7.1.2. Precision and Accuracy Assessment

Rigorous assessment of precision and accuracy across all target analytes ensures reliable quantitative performance and demonstrates method robustness for large-scale epidemiological applications [134,135]. Analytical precision, quantified as the relative standard deviation of intra- and inter-batch variability, was less than 20% [12]. The 1 ng/mL spiking quality control pool exhibited analyte recoveries between 83% and 109% efficiency, with a median of 97% efficiency [12]. The inter-batch precision (range, median) exhibited a relative standard deviation (RSD) of 0–18% (3.5%) and a coefficient of variation (CV) of 2–19% (9.5%) [12]. Quality assurance/control (QA/QC) tests indicated that analyte recoveries from spiking tests varied between 72% and 128%, matrix effects ranged from 74% to 119%, inter-batch coefficients of variation were below 20% (with 50 samples per batch), and there was no evidence of background contaminants in field and procedural blanks (one blank processed alongside ten samples) for the target analytes [38]. The approach exhibited adequate recovery (81–120%) for the majority of the added analytes, with acceptable relative standard deviations (<20%) across three spiking levels [27]. The accuracy for all compounds was between 85% and 115%, and the precision was less than 15% [13]. The relative standard deviations for intra- and inter-day precision ranged 0.1–16.4% and 0.3–18.9% for plasma, while for urine, they ranged from 0.1 to 16.1% and from 1.5 to 19.6%, respectively, remaining below the 20% acceptability threshold set by the FDA [14]. These precision and accuracy results demonstrate that multiclass methods can achieve performance standards suitable for quantitative exposome research while maintaining reliability across hundreds of target analytes (Table 3) [134,135].

### 7.2. Matrix Effect Evaluation

Comprehensive evaluation of matrix effects across diverse biological and environmental matrices is essential for ensuring accurate quantification and understanding the impact of sample composition on analytical performance [136,137,138,139,140,141]. Matrix effects provide significant problems for LC-MS analysis, arising from the co-elution of sample matrices with analytes during liquid chromatography separation [39]. The matrix effects from urine were more pronounced than those from the sewage effluent, likely due to the higher concentrations of inorganic salts, urea, and creatinine present in urine matrices [39]. Matrix effects were mitigated through the utilization of matrix-matched calibration standard curves and stable isotope-labeled internal standards [12]. Signal suppression or enhancement was evaluated as the ratio of the slope of the calibration curve generated in a urine matrix to that of a curve developed in reagent-based water [12]. The SSE of the 50 analytes in this investigation ranged from 0.8 to 1.2, a ratio that was deemed suitable [12]. The majority of veterinary drugs, pesticides, and antibiotics (69%) showed matrix effects within a range of 50–140% [27]. The matrix effects were quantified, ranging from −18% to 63%, indicating either suppression or augmentation of ESI-MS/MS signals for the target compounds. To alleviate such matrix effects, structurally analogous and stable isotope- labeled compounds were utilized as internal standards [30]. Matrix effects at concentrations of 0.5 and 5 ng/mL were predominantly minimal for the phospholipid-free matrix, exhibiting medians of 91% and 107%, respectively [34]. Systematic evaluation of matrix effects demonstrates that multiclass methods can achieve acceptable performance across diverse sample types through appropriate use of internal standards and matrix-matched calibration approaches [136,137,138,139,140,141].

### 7.3. Reference Materials and Standardization

The use of certified reference materials and involvement in proficiency testing programs provide external validation of technique efficacy and guarantees traceability and comparability of results among laboratories and research [62,142,143,144]. The outcomes from the optimized multi-class technique were validated in formal international proficiency testing programs [12]. The procedure performed satisfactorily; the submitted findings generally fell within the tolerance range of the proficiency testing reference values, and were therefore deemed validated [12]. Additionally, standard reference materials (SRMs) (SRM 3672 [145] and SRM 3673 [146]; National Institute of Standards and Technology) and three aliquots each of HHEAR urine quality control (QC) pools A and B were analyzed in every batch [32]. The SRMs contained 20 analytes, but mean recoveries were calculated for 19, excluding one analyte with a spiked level below the LOD, ranging from 70 to 129% [32]. The testing of SRM 1957 [147] indicated that the mean concentrations of selected compounds (PFAS) varied between 89.6% to 110.2% in accordance with established levels, thereby confirming the analytical precision [28]. Six analytes having certified reference values in NIST SRM 3672 (Organic Contaminants in Smokers’ Urine) were identified and measured, exhibiting a relative error of less than 20% compared to the reference value for all analytes [22]. The laboratory engages in external proficiency testing programs, including the German External Quality Assessment Scheme (G-EQUAS) for biological material analyses and the Organic Substances in Urine Quality Assessment Scheme (OSEQAS) administered by the Centre de Toxicologie du Quebec in Canada, to validate method performance and ensure result quality [12,29]. The successful performance in proficiency testing programs and accurate quantification of certified reference materials demonstrate the reliability and traceability of multiclass analytical methods, providing confidence in their application to large-scale exposome studies and regulatory monitoring programs [62,142,143,144]. Standardization efforts through proficiency testing programs and certified reference materials represent essential components for ensuring data quality and comparability across laboratories conducting multiclass exposome analysis. The successful performance in international proficiency testing programs demonstrates method reliability and enables harmonized data integration across different analytical platforms and study populations. These standardization approaches are particularly critical for large-scale exposome-wide association studies where data from multiple laboratories must be integrated to achieve sufficient statistical power and population representation for meaningful health risk assessment.

### 7.4. Practical Solutions for Standardizing Methods Between Different Laboratories

Standardization of multiclass analytical methods across laboratories requires a comprehensive approach that encompasses multiple quality assurance elements to ensure reproducible and comparable results. Participation in formal international proficiency testing programs represents a cornerstone of method standardization, as demonstrated by successful qualification of multiclass methods in established programs where submitted results typically fall within tolerance ranges of reference values, thus validating analytical performance. Implementation of certified reference materials provides essential benchmarks for method validation, with studies showing accurate quantification of target analytes in standard reference materials such as SRM 1957 for PFAS analysis and SRM 3672 for organic contaminants in smokers’ urine. Standardized quality control protocols should include the regular analysis of quality control pools and standard reference materials in each analytical batch. Established acceptance criteria should be set for recovery (for example, 70–130%), matrix effects (for example, 70–130%), and inter-batch coefficients of variation (for example, <20%). Laboratory participation in external quality assessment schemes such as the German External Quality Assessment Scheme (G-EQUAS) for biological materials and the Organic Substances in Urine Quality Assessment Scheme (OSEQAS) and AMAP Ring Test for Persistent Organic Pollutants in Human Serum (AMAP) conducted by the Centre de Toxicologie du Quebec provides ongoing validation of method performance and ensures result quality. Adoption of standardized sample preparation protocols utilizing consistent extraction methods, such as optimized solid-phase extraction with 96-well plates or standardized liquid–liquid extraction procedures with defined solvent compositions and extraction cycles, ensures reproducible recovery across laboratories. Implementation of uniform analytical parameters including standardized multiple reaction monitoring transitions, optimized collision energies, and consistent chromatographic conditions enables comparable sensitivity and selectivity across different laboratory platforms. Establishing common validation criteria that address the unique challenges of multiclass methods is essential. This includes defining acceptable ranges for extraction recovery (for example, 70–130%), precision (for example, RSD < 30%), and sensitivity requirements (such as sub-ng/mL detection limits). These criteria provide consistent performance benchmarks.

## 8. Health Implications and Exposure-Response Relationships

Multiclass exposome approaches have enabled groundbreaking exposome-wide association studies that identify environmental chemical risk factors for human health outcomes, demonstrating the critical importance of comprehensive chemical exposure assessment in understanding disease etiology. A notable example is the nested gestational diabetes mellitus (GDM) case–control study where multiclass analysis of 325 chemicals revealed significant associations between chemical mixture exposure and GDM risk [28]. The Bayesian kernel machine regression analysis revealed a robust linear positive correlation between co-exposure to 33 chemicals and the risk of gestational diabetes mellitus (GDM). The joint effect was statistically significant when exposure levels of all chemicals were between the 25th and 75th percentiles. In comparison to the 50th percentile, the predicted probit of GDM prevalence at the 75th percentile indicated a 92% increase [28]. The research determined that significant contributions to the combined impacts originated from PFAS, as indicated by the group posterior inclusion probability (groupPIP) of 1.00, synthetic antioxidants with groupPIP of 1.00, and non-PAE plasticizers with groupPIP of 0.80, with key chemicals including PFHxS with conditional PIP (condPIP) of 0.64, DPG with condPIP of 1.00, and DBF with condPIP of 0.91 driving the joint effect [28]. Similarly, hair analysis studies in Chinese women revealed that exposure to multiclass organic contaminants correlated with altered sex steroid hormone levels, with each hormone linked to a mixture of at least 10 pollutants. Notably, hair E2 concentration was associated with 19 xenobiotics, including propoxur, γ-hexachlorocyclohexane, fipronil, permethrin, prochloraz, mecoprop, and carbendazim [33]. Further ExWAS applications encompass the CIRCA CHEM chrononutrition trial, which expanded from two pesticide biomarkers to 125 biomarkers of exposure to food contaminants utilizing exposomics tools. This study unveiled significant positive correlations between oxidative stress biomarkers HNEMA and F2A8IP and a range of urinary biomarkers indicative of exposure to various xenobiotics, which includes VOCs, flame retardants, phthalates, pesticides, tobacco metabolites, PAHs, and phytoestrogens [29]. Longitudinal plasma exposome studies demonstrated mixed-effect modeling with statistically significant interactions between the exposome and metabolome, suggesting endocrine disruption. Positive correlations were identified between testosterone and various PFAS, including perfluorononanoate, linear PFOS, and perfluorodecanoate in females, with these associations maintaining statistical significance in adjusted models accounting for baseline age and BMI [35]. These ExWAS examples illustrate how multiclass analytical approaches enable the identification of complex exposure-health relationships that would be missed by traditional single-analyte methods, providing critical evidence for environmental health risk assessment and regulatory decision-making while supporting the development of precision medicine approaches for environmental health protection.

## 9. Current Challenges and Limitations

### 9.1. Analytical Challenges

#### 9.1.1. Chemical Diversity and Physicochemical Properties

**Figure 8:**
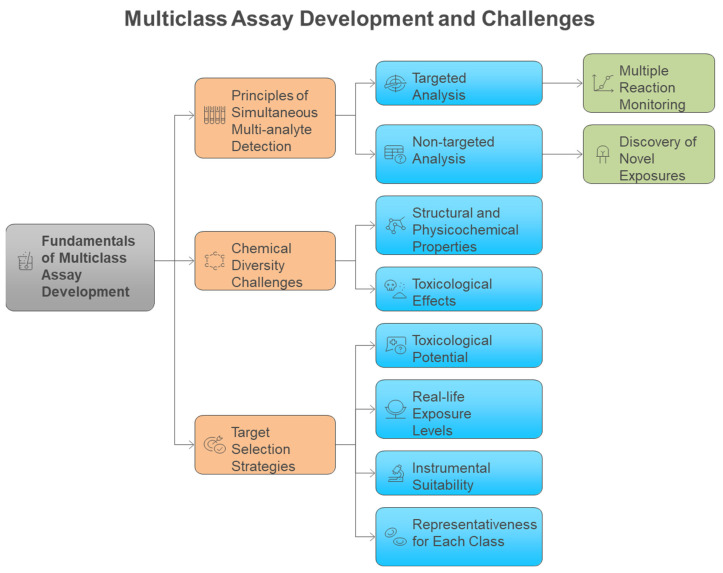
Multiclass Assay Development and Challenges in Environmental Chemical Exposome Analysis.

The vast chemical diversity and physicochemical properties of environmental contaminants present fundamental challenges for simultaneous analysis, requiring analytical methods that can accommodate compounds spanning orders of magnitude in polarity, volatility, and molecular weight [22,41]. The diverse structural and physicochemical characteristics of toxicants present a significant difficulty for the simultaneous investigation of many chemical classes [16]. The diverse chemical characteristics of multiclass CECs provide significant challenges for simultaneous investigation [39]. The significant variation in physical and chemical features, including a broad polarity range, poses a considerable difficulty for concurrent screening via a single LC-MS study [37]. The extensive physicochemical array of xenobiotics suggests a broad spectrum of toxicological consequences on humans, including liver carcinogenicity, nephrotoxicity, and estrogenicity [22]. Approximately 5000 environmental chemicals are estimated to be distributed and deposited in people, and the chemical residues can be converted into several product forms by phase-I and phase-II processes including dealkylation, hydroxylation, and oxidation in biological metabolism, hence broadening their characteristics and the screening list of exogenous mixes [37]. The integrated and thorough assessment of the metabolome and exposome is complex due to the amounts of metabolites, pharmaceuticals, dietary components, and environmental pollutants varying over nearly 10 orders of magnitude and encompassing a wide range of chemical classes [22]. The presence of target analytes with diverse physical and chemical characteristics, including polarity, poses a significant problem for solid-phase extraction (SPE) procedures in exposomics, owing to the different interactions between analytes and sorbents. The enormous chemical diversity of the exposome necessitates continued development of analytical strategies that can accommodate the full spectrum of environmental contaminants while maintaining analytical performance [41]. Figure 8 illustrates the comprehensive framework for multiclass assay development, highlighting the dual approach of targeted multiple reaction monitoring and non-targeted discovery methods. The schematic emphasizes critical selection criteria including toxicological potential, real-life exposure levels, instrumental suitability, and class representativeness essential for effective exposome characterization.

#### 9.1.2. Sensitivity Requirements for Trace Analysis

Sensitivity requirements for trace analysis in exposome research demand detection capabilities at ultra-low concentration levels, often in the presence of complex matrices containing endogenous compounds at concentrations orders of magnitude higher than target analytes [34,93]. Exposure compounds, generally found at trace concentrations, are primarily measured via targeted LC-MS/MS [24]. Nonetheless, numerous current methodologies are constrained to a limited spectrum of analyte categories or exhibit inadequate sensitivity for exposomic analysis, and their applicability to extensive sample cohorts for exposome-wide association studies (ExWAS) has yet to be validated [51,62]. This is especially important when analytes are present at trace levels [24]. The substantial number of analytes per test and the necessary ultimate technique sensitivity in the pg-ng/mL range necessitate tailored adjustments of analytical figures of merit [24,34]. The concentrations of exogenous residues in serum are typically 100–1000 times lower than those of endogenous metabolites, resulting in insufficient fragments of foreign residues being acquired through repeated DDA during a single injection screening [37]. The problem of chemical exposomics in human plasma is the thousand-fold concentration disparity between endogenous compounds and external contaminants [34]. Enhancing the sensitivity of chemical exposomics can be achieved by increasing the introduction of environmental analytes on-column, either by preconcentration methods or large-volume injection [34]. In practice, however, this can lead to increased interference and instrumental fouling from major endogenous substances [34]. These extreme sensitivity requirements push the limits of current analytical technology and necessitate continued innovation in sample preparation and instrumental methods [41,51].

### 9.2. Biological and Environmental Complexity

Biological and environmental complexity presents multifaceted challenges for exposome research, including matrix effects, temporal variability in exposure patterns, and the need to account for complex exposure scenarios across diverse populations and environments [17,41,51]. Matrix effects provide significant problems for LC-MS analysis, arising from the co-elution of sample matrices with analytes during liquid chromatography separation [39]. Additionally, matrix components might affect the trapping efficiency of the ionic CECs on the trap column [39]. The matrix effects from urine were more pronounced than those from the sewage effluent, likely due to the higher concentrations of inorganic salts, urea, and creatinine present in urine matrices [39]. Numerous highly polar molecules were lost during the SPE process due to inadequate retention with the sorbent, and significant matrix effects were identified for various analytes [17]. Nonetheless, the EC criteria offer restricted relevance for exposome-scale LC-MS/MS methodologies due to the intricate nature of extensive multianalyte techniques and the exceedingly low concentrations of the majority of exposure chemicals in human matrices [24]. The EC criteria lack definitive thresholds for precision and repeatability about analyte concentrations under 120 µg/kg and fail to consider the extensive array of analytes in extreme multianalyte methodologies essential for exposomics [24]. The constraint on sample quantity is particularly relevant to more intrusive sample types, such as blood, where sample quantities typically range from a few hundred microliters for each individual, and may be considerably lower depending on the research design and analytical methods employed for each sample [19]. The complexity of biological and environmental systems requires sophisticated analytical and statistical approaches to account for matrix variability and temporal dynamics in exposure assessment [41,51,118].

### 9.3. Data Integration and Interpretation

Data integration and interpretation represent major bottlenecks in exposome research, with challenges including the vast amount of unidentified molecular features, limited spectral databases, and the need for advanced computational approaches to extract meaningful biological insights from complex datasets [47,51,148]. The predominant number of untargeted molecular features remains uncharacterized, necessitating the enhancement of laboratory methodologies and data analytics for more extensive studies [35]. The anticipated task of elucidating the impact of the chemical exposome on disease progression necessitates the application of both network science and systems biology to combine chemical and biomolecular relationships across various omics levels; however, the inherent instability of the chemical exposome presents a significant practical obstacle to this methodology [35]. Furthermore, the approach currently lacks direct indicators of biological action [16]. The restricted quantity of chemicals identified in metabolomics databases impedes their annotation and subsequent biological interpretation [14]. It is important to recognize that standards were not accessible for numerous metabolites that were tested [14]. Although the methodology encompasses 325 CECs, certain ones may not serve as adequate indicators of internal exposure. Additionally, for numerous target CECs, the biotransformation kinetics and principal metabolites remain ambiguous, hence constraining our capacity to find effective exposure markers for biomonitoring [28]. This partially leads to low detection rates or the absence of detection of numerous CECs in humans [28]. The challenges of data integration and interpretation highlight the need for improved computational tools, expanded chemical databases, and better understanding of exposure biomarker relationships to fully realize the potential of exposome research for human health protection [47,51,149,150].

### 9.4. Practical Considerations for Selecting Analytical Methods

The selection of appropriate multiclass analytical methods requires careful consideration of cost, time, and resource requirements, particularly for novice researchers entering the field of exposome analysis. Liquid–liquid extraction approaches offer cost-effective solutions with broad chemical coverage, though they may require additional cleanup steps, while solid-phase extraction methods, despite higher initial costs for sorbents and automation equipment, provide superior cleanup capabilities and enhanced selectivity suitable for large sample sizes and high throughput applications [12,17]. Triple quadrupole mass spectrometry systems, while representing significant capital investment, deliver exceptional sensitivity with limits of detection in the 0.001–0.1 ng/mL range and provide the quantitative precision essential for comprehensive exposome studies [12,24,29]. Large volume injection techniques can enhance method sensitivity without extensive sample preconcentration, though they require careful optimization to manage matrix effects and may necessitate discarding initial column eluate [39]. The time investment varies considerably, with targeted methods employing optimized multiple reaction monitoring requiring extensive upfront optimization of mass spectrometric parameters for each analyte, while non-targeted approaches demand substantial computational resources and expertise for data processing and compound identification [11,24].

### 9.5. Ethical and Sampling Considerations for Invasive Procedures

Multiclass exposome analysis involving invasive sampling procedures requires careful consideration of ethical and practical constraints. Cerebrospinal fluid collection necessitates lumbar puncture procedures, making samples particularly precious due to their invasive nature and the methodological challenges associated with relatively low chemical concentrations compared to other matrices like blood. Pediatric populations present additional ethical considerations, as demonstrated by the recent expansion of NHANES protocols to include urine collection from children aged 3–5 years following validation of collection feasibility in young age groups. The vulnerability of early life stages to environmental chemical exposures necessitates expanded biomonitoring efforts while maintaining appropriate ethical safeguards for these populations.

## 10. Future Directions and Emerging Trends

### 10.1. Technological Advances in Mass Spectrometry

**Figure 9:**
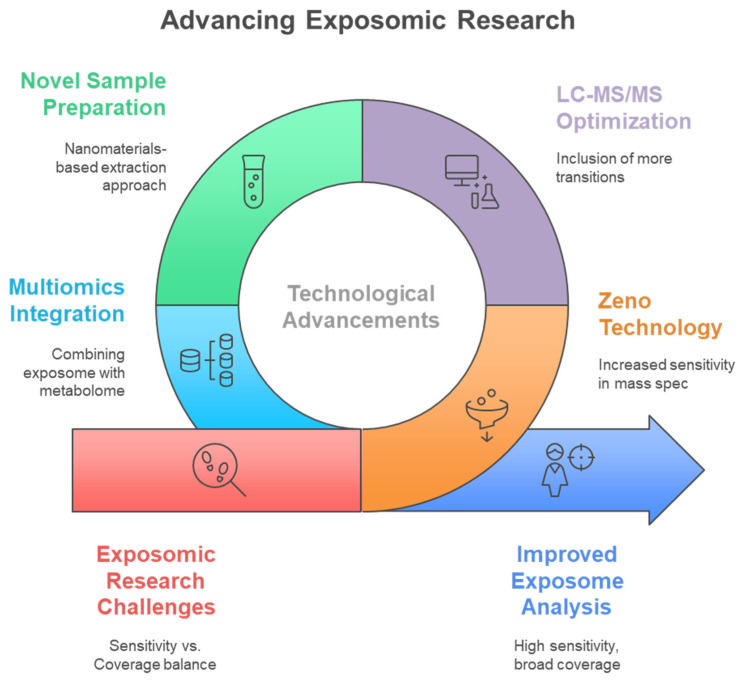
Future Directions and Technological Advances in Multiclass Exposome Analysis.

Technological advances in mass spectrometry continue to drive improvements in sensitivity, selectivity, and throughput for multiclass exposome analysis, with emerging technologies offering unprecedented capabilities for comprehensive chemical characterization [25,41,151]. While nontargeted, high-resolution approaches are considered as the pivotal technology for exposomics research in the future [41,51], presently, targeted techniques utilizing triple quadrupole instrumentation continue to offer superior sensitivity, linearity and reduced data processing requirements [16]. The implementation of the newly developed Zeno technology, a linear ion trap preceding the TOF analyzer that facilitates duty cycles of up to 90%, leads to substantial enhancements in sensitivity [25]. This study offers a promising solution to a significant challenge in the small-molecule omics domain: achieving a balance between high specificity and extensive chemical coverage. It has been demonstrated in exposomic applications but may be applicable to lipidomics and metabolomics workflows [25]. The LC-MS/MS system is not yet operating at full capacity, as it retains the potential for the incorporation of hundreds of more mass spectrometric transitions [16]. For instance, coupling it to a high-resolution MS instrument can be applied in nontarget screening of a wide range of CECs in aqueous samples [39]. Through ongoing advancements in sample purification, chromatography, and mass spectrometry technologies [17,41], further advancements in multi-class methodologies will likely emerge to address existing obstacles and enhance the research of the human exposome [12,29]. These technological advances promise to further expand the capabilities of multiclass methods while addressing current sensitivity limitations [41,51]. Figure 9 illustrates the convergence of emerging analytical technologies and methodological innovations that are driving the evolution of multiclass exposome analysis toward more comprehensive, sensitive, and integrated approaches for environmental health research. The schematic illustrates key technological developments and research priorities for advancing comprehensive environmental chemical exposure measurement, including novel sample preparation approaches, mass spectrometry optimization, multiomics integration, and the ongoing challenge of balancing analytical sensitivity with broad chemical coverage in exposome-wide association studies.

### 10.2. Novel Sample Preparation Approaches

Novel sample preparation approaches represent a critical frontier for improving multiclass method performance, with emerging techniques offering enhanced selectivity, reduced matrix effects, and improved automation capabilities for large-scale studies [17,41,85,86,152,153,154,155]. Additional endeavors will be pursued to enhance the LLE process by experimenting with alternative solvent combinations or to augment extraction efficiency through the use of nanomaterials-based techniques [28]. The LLE method is compatible with numerous CECs; nonetheless, it may yield suboptimal recoveries or significant matrix effects for certain chemicals. By utilizing appropriately isotopically labeled compounds, we can implement effective adjustments in most instances [28]. Integrating many orthogonal methodologies may mitigate these constraints; nevertheless, it would be both time-intensive and expensive [24]. Exposome-type assays must achieve a well-balanced compromise among analyte coverage, effective sample cleanup, assay sensitivity, and time constraints [24]. Notably, there are enormous potentials for this “all-in-one” method with a mixed-mode trap column [39]. Future endeavors will encompass the inclusion of other toxicologically pertinent compounds and the evaluation of the proposed sample preparation workflow’s efficacy in extensive cohort studies to assess its influence on instrumental reliability and operational downtimes [24]. These novel approaches to sample preparation will enable more comprehensive chemical recovery while reducing analytical complexity and improving method robustness [17,41,85,86,152,153,154,155].

### 10.3. Integration with Other Omics Technologies

Integration with other omics technologies represents a transformative approach for exposome research, enabling comprehensive understanding of exposure-response relationships through simultaneous measurement of chemical exposures and biological responses [117,156,157,158]. In longitudinal exposome investigations, this may be approached via multiomics; however, the comparative dynamics of every molecular dataset must be taken into account during the study design phase [35]. The results underscore the necessity of conducting multiple assessments of the chemical exposome in epidemiology studies to reduce exposure misclassification. In longitudinal multiomics research, the exposome should be evaluated as often, or more often, than other biomolecular profiles [35]. The novel exposomics approach complements metabolomics methods, utilizes open science resources, and is scalable to facilitate extensive research of the exposome [34]. The application of both approaches to identical samples may be constrained by sample sizes, as well as concerns of cost and analysis duration [34]. The chemical exposomics approach outlined below is not intended to supplant metabolomic analysis in human investigations; rather, it offers distinct advantages in method sensitivity, facilitating the detection, quantification, or discovery of trace analytes inside the exposome [34]. This methodology in metabolomics can be utilized to identify weakly ionized and/or low-concentration chemicals, and scenarios with restricted sample availability may also gain advantages, enabling both targeted and untargeted data collecting in a single injection [25]. Integration of exposomics with metabolomics, genomics, and other omics platforms will provide unprecedented insights into the mechanisms underlying exposure-health relationships (Figure 9) [117,156,157,158].

### 10.4. Artificial Intelligence and Machine Learning Applications

Artificial intelligence and machine learning applications offer revolutionary potential for exposome research, enabling advanced pattern recognition, predictive modeling, and automated data interpretation across complex multi-dimensional datasets [47,159,160,161]. N-Butylbenzene sulfonamide, 2-tert-butylphenol, and 2-naphthol were chosen from a list of xenobiotics, rated by their similarity to established estrogen receptor agonists, for the initial application of cognitive computing and artificial intelligence in exposome research [11]. These compounds were chosen due to their high scores and the absence of experimental reports on their estrogenicity to date [11]. The exposomics techniques utilized comprised numerous biomarkers of exposure and impact (exceeding 120 in total), a multi-class biomarker test inside a single sample, and the processing and visualization of exposome-wide association research data alongside chrono-metabolism outcomes [29]. The CIRCA CHEM chrononutrition trial aimed to extend and replicate previous findings from two pesticide exposure biomarkers to 125 food contaminant/xenobiotic exposure biomarkers, employing exposomics methodologies, including exposome-wide association evaluation and various machine learning models, such as exposome-based visualization techniques [29]. The application of artificial intelligence and machine learning approaches will accelerate discovery of novel exposure–health relationships and enable more sophisticated analysis of complex exposome datasets, ultimately supporting precision medicine and targeted public health interventions (Figure 9) [160,161,162].

## 11. Conclusions and Recommendations

The current state of multiclass assay technology represents a significant advancement in exposome research capabilities, with validated methods now enabling simultaneous quantification of hundreds of environmental chemicals across diverse biological matrices. However, several fundamental scientific questions and methodological challenges emerge from our comprehensive analysis of literature that require urgent attention in future exposome research. The most pressing technical challenge involves resolving the inherent trade-off between analytical sensitivity and comprehensive chemical coverage, as exposome-type assays must achieve a balanced compromise among analyte coverage, effective sample cleanup, assay sensitivity, and time constraints. Current methods face significant limitations in simultaneously achieving the ultra-trace sensitivity required for environmental contaminants while maintaining broad chemical coverage across the vast physicochemical diversity of the exposome.

A critical gap exists in computational infrastructure and chemical identification capabilities, a multitude of untargeted molecular properties remain uncharacterized, necessitating the enhancement of laboratory methodologies and data analysis for more extensive studies. The restricted quantity of compounds identified in metabolomics databases impedes annotation and subsequent biological interpretation, while several target chemicals may be inadequate markers for internal exposure due to ambiguous biotransformation kinetics and predominant metabolites. This essential information deficiency constrains our capacity to identify suitable exposure markers for biomonitoring, leading to low detection rates or the absence of detection for several substances in people. Methodological standardization constitutes a vital research requirement, as existing validation criteria offer restricted applicability for exposome-scale methodologies due to the intricate nature of large-scale multianalyte techniques and the exceedingly low levels of most exposure compounds in human matrices. The criteria lack explicit thresholds for precision and repeatability about analyte concentrations and fail to consider the extensive array of analytes in complex multianalyte methodologies essential for exposomics. Future research must create customized validation frameworks specifically intended for exposome-scale applications. The integration of exposome data with various omics platforms offers both prospects and obstacles, as elucidating the impact of the chemical exposome upon disease progression necessitates network science and systems biology methodologies to synthesize chemical and biomolecular connections across multiple omics tiers. The very low stability of the chemical exposome is a significant practical obstacle to this methodology, underscoring the necessity of repeated tests in epidemiological research to reduce exposure misclassification.

Critical research needs in multiclass exposome analysis encompass both technical improvements and expanded applications, requiring continued innovation to address current limitations while extending analytical capabilities to new chemical classes and biological matrices. Recommendations for future development emphasize the need for integrated approaches that combine technological innovation with systematic implementation in large-scale studies, ultimately supporting the translation of exposome research into actionable public health interventions.

## Figures and Tables

**Table 1:** Overview of Multiclass Assays for Environmental Chemical Mixtures Exposure Measurement in Humans: Study Characteristics, Analytical Platforms, and Performance Metrics.

Study	Study Design	Sample Information	Analytical Platform	Number of Biomarkers	Study Duration	Primary Objective
Braun, et al. [19]	Method comparison study	Pooled human plasma	LC-HRMS and GC-HRMS	>400 chemicals	Single timepoint	Compare extraction efficacy of three methods for chemical recovery and bioassay compatibility
Engelhardt, et al. [20]	Method development	30 serum samples	LC-MS/MS, GC-MS	37 target analytes	Not specified	Develop multi-target analytical methods for synthetic phenolic compounds
Fareed, et al. [21]	Comparative study	Breast milk, urine, plasma samples	LC-MS/MS	>80 xenobiotics	Not specified	Evaluation of phase II biotransformation in human biofluids. Compare deconjugation efficiencies of different enzymes
Flasch, et al. [22]	Longitudinal	77 urine samples	LC-HRMS	145 endogenous metabolites + 106 xenobiotics	Not specified	Comprehensive analysis of endogenous metabolome and chemical exposome
González-Domínguez, Jáuregui, Queipo-Ortuño and Andrés-Lacueva [14]	Method validation	10 volunteers	LC-MS/MS	>1000 metabolites	1 month intervention	Develop multianalyte metabolomics platform for exposome research
[23]	Method development	Not specified	LC-MS/MS, LC-HRMS	94 diverse analytes	Not specified	Develop high-throughput SPE protocol for targeted and nontargeted exposomics
Gu, et al. [24]	Method development and validation	200 urine samples from 50 pregnant women	LC-MS/MS with SPE in 96-well plates	>230	Not specified	Develop scalable workflow for analyzing biomarkers in urine, plasma, and serum
Hernandes and Warth [25]	Method development	SRM 1950 and SRM 1958	LC-HRMS with Zeno technology MRM-HR + SWATH	135 toxicants	Not specified	Combined targeted/untargeted LC-MS method for exposomics
Hernandes, et al. [26]	Method optimization, Proof of principle for 12 compounds	6 donors	LC-HRMS	>200 xenobiotics	6 months storage	Optimize LC-HRMS workflow for combined exposomic and metabolomic analysis in DBS
Hossain, et al. [27]	Method expansion	Not specified	LC-MS/MS	>120 xenobiotics	Not specified	Scale up targeted exposome method by incorporating veterinary drugs and pesticides
Huang, et al. [28]	Nested case–control	360 plasma samples (120 GDM cases, 240 controls)	LC-MS/MS	325 CECs	Not specified	Target exposome for gestational exposure characterization
Jagani, Pulivarthi, Patel, Wright, Wright, Arora, Wolff and Andra [12]	Method development and validation	15 urine samples	UHPLC-MS/MS	50	Not specified	Develop multi-class method for quantitation of biomarkers in urine
Jamnik, Flasch, Braun, Fareed, Wasinger, Seki, Berry, Berger, Wisgrill and Warth [16]	Method development and application	21 extremely premature infants, 86 breast milk samples	LC-MS/MS with protein precipitation	>80	Infants < 28 days, Breast milk; 211 days post-partum	Develop sensitive LC-MS approach for xenobiotics analysis
Kunde, et al. [29]	Randomized cross-over chrononutrition trial	45 participants	UHPLC-MS/MS	125 biomarkers	14 days plus washout	Determine effect of time restricted eating on biomarkers of exposure to food contaminants and chrono-metabolism patterns
Lee, Lee, Han, Lee, Sung, Min, Im, Han, Cha and Lee [13]	Case–control	39 urine samples (19 mother-newborn pairs)	LC-ESI-MS/MS	86 EPOLs	Not specified	Multiple exposure assessment of multiclass environmental pollutants
Lin, et al. [30]	Cross-sectional	20 urine samples	HPLC-MS/MS	35 phenolic compounds	Not specified	Simultaneous determination of multiple phenolic compound classes
Marchiandi, et al. [31]	Preconception cohort study	30 couples	LC-MS/MS with protein precipitation	95	Not specified	Characterize chemical exposome in paired human preconception pilot study
Oh, et al. [32]	Cross-sectional pilot study	201 children aged 2–4 years	LC-MS/MS	111	Not specified	Assess chemical exposures in young children using ECHO Cohort
Peng, et al. [33]	Cross-sectional	196 women	LC-MS/MS, GC-MS/MS	54 pollutants, 9 hormones	Not specified	Evaluate relationship between multiclass organic pollutants and sex steroid hormones
Preindl, Braun, Aichinger, Sieri, Fang, Marko and Warth [11]	Proof-of-principle	Urine (6) serum, breast milk (9) samples	LC-MS/MS	75 xenoestrogens	Not specified	Generic method for xenoestrogen determination in biological matrices
Sdougkou, et al. [34]	Method validation	34 plasma samples	LC-HRMS	77 priority analytes	Not specified	Validate phospholipid removal protocol for chemical exposomics
Sdougkou, et al. [35]	Longitudinal cohort study	46 adults, 6 visits each	LC-HRMS	83 targeted + 519 annotated	2 years	Apply high-resolution chemical exposomics to plasma
Talavera Andújar, et al. [36]	Pilot study	30 CSF samples	LC-HRMS	>1000 metabolites, Targeted quantification of 35 bile acids	Not specified	Complement traditional AD biomarkers with small-molecule analysis
Wang, et al. [37]	Cross-sectional Screening study by spiking the pooled samples	24 pooled serum samples	2D-LC-HRMS	1210 exogenous chemicals	Not specified	Screening strategy for exogenous chemicals in serum using online 2D-LC-HRMS & Full MS/DIA MS^2^ (i.e., MS/MS)
Zhang, et al. [38]	Cross-sectional study	180 outpatients	UHPLC-Orbitrap MS & MS/MS	28	Not specified	Develop suspect screening strategy for environmental chemicals in CSF
Zhao, et al. [39]	Method development	Various water and urine samples	LC-MS with column-switching	102	Not specified	Develop LC-MS method for simultaneous analysis of wide range of multiclass CECs

Abbreviations: 2D-LC-HRMS, Two-Dimensional Liquid Chromatography-High-Resolution Mass Spectrometry; CECs, Contaminants of Emerging Concern; CSF, cerebrospinal fluid; DBS, dried blood spots; ECHO, Environmental influences on Child Health Outcomes; EPOLs, Environmental Pollutants; GC-MS, Gas chromatography-mass spectrometry; GC-HRMS, Gas Chromatography-High-Resolution Mass Spectrometry; GDM, Gestational Diabetes Mellitus; LC-ESI-MS/MS, Liquid Chromatography–Electrospray Ionization–Tandem Mass Spectrometry; LC-HRMS, Liquid Chromatography–High-Resolution Mass Spectrometry; LC-MS/MS, Liquid Chromatography–Tandem Mass Spectrometry; SPE, solid-phase extraction; SRM, Standard Reference Material; SWATH, Sequential window acquisition of all theoretical fragments; UHPLC, ultra-high-performance liquid chromatography.

**Table 2:** Mass Spectrometry Platforms, Sample Preparation and Chromatographic Separation Strategies, and Method Validation Parameters for Multiclass Analysis of this Review.

MS Platform	Sample Preparation Strategy	Chromatographic Approach	Sample Volume (μL)	Matrix Effects (%)	Precision (RSD%)	Method Validation	Quality Assurance	Study
LC-HRMS and GC-HRMS Q Exactive orbitrap	3 extraction protocols comparison PES + SPE SPE alone Solvent Precipitation	DB5-ms GC Column Acquity BEH C18 column	300	Not specified	CV < 40% for recoveries, <30% for standards	Internal standard calibration	Quality criteria applied	[19]
TSQ 9000, Xevo TQ-S Micro	Protein precipitation with ACN + SPE	DB-35MS UI, ACE C18-PFP	0.5 g	Not specified	11% average	Recovery, precision, reproducibility	Matrix-matched calibration RSD of Pooled QC, CRM	[20]
QTRAP 6500+	Enzymatic hydrolysis + LLE	RP-LC	50–200	Variable	Not Specified	Hydrolysis efficiencies of different enzymes were checked based on signal using a Conjugate reference mixture	Matrix matched calibration, Matrix spikes and blanks	[21]
Q Exactive HF Quadrupole-Orbitrap	LLE with ACN/MeOH	RP + HILIC dual column	200	Variable	22–31% median RSD	Recovery 74–124%	SRM validation	[22]
QTRAP 6500	Protein precipitation (Plasma) Dilution (Urine)	Luna Omega Polar C18	20 (urine), 100 (plasma)	Majority Negligible	<20%	Linearity, recovery, matrix effects, precision (CDC, FDA)	CVs of Internal standards concentration, peak width, RT	[14]
QTRAP 6500+	SPE with Oasis HLB in 96-well plates	HSS T3 column	400	60–140% acceptable	Not Specified	Recovery, matrix effects, linearity	Spiked pools and NIST	[23]
QTRAP 7500	SPE with Oasis HLB in 96-well plates	HSS T3 column	400	SSE within 60–130%	<30	New validation framework for exposomics	Multiple QC levels	[24]
ZenoTOF 7600	Protein precipitation	RP-LC	30	Variable	Variable	Matrix-matched calibration	Labeled IS correction	[25]
ZenoTOF 7600	Liquid extraction with ACN/MeOH/water	HSS T3 column	50 (DBS)	76% median	18% median	Recovery, matrix effects, LOD estimation	Pooled QC, blank samples	[26]
QTRAP 6500+	Protein precipitation with ACN/MeOH	HSS T3 column	200	50–140% acceptable	<20%	Linearity, accuracy, precision, LOD/LOQ EC 2002, Eurachem 2014	Matrix matched calibration, Pooled QC	[27]
Triple Quad 7500	LLE with ethyl acetate/n-hexane	RP-LC	200	Mean matrix effect 50–150%	<30%	Extraction Efficiency: 50–150%, Matrix effect, Intra & Inter batch variation	IS normalized calibration curve, Pooled QC, SRM 1957 validation	[28]
Triple Quad 6500+	Enzymatic deconjugation + SPE with Oasis HLB	Three separate LC injections	200	Signal suppression or enhancement (SSE) 0.8–1.2 ratio	<20	Proficiency testing qualification	Multiple QC pools and blanks	[12]
QTRAP 6500+	LLE + Protein precipitation with ACN/MeOH	HSS T3 column	200–250	Variable	16–32%	European Commission Decision No. 657/2002	Isotope-labeled internal standards, Matrix matched calibration	[16]
Triple Quad 6500+	Enzymatic deconjugation, SPE cleanup	Three LC columns: Hypersil Gold AQ, Betasil C18, Kinetex C8	200	Not specified	<20%	80–110% extraction recovery	G-EQUAS and OSEQAS proficiency testing	[29]
TSQ Altis QQQ	Tandem hybrid hydrolysis	RP-LC with comprehensive mobile phase	200	Not specified	<15%	Accuracy 85–115% Brodie & Hill Guidance, ICH Q2B guidelines	SRM validation	[13]
Triple Quad 6470	Enzyme hydrolysis + SPE	C18-RP LC	2000	18–63%	<20%	Spike recovery	ISTD recovery and application to urine samples	[30]
Triple Quad 6495 C	Enzymatic pretreatment and Protein precipitation with ACN	Zorbax C18 column	100	Majority 71–110	Limit set to 25%	ICH Q2(R1) and EC 2002/657/EC	Matrix-matched calibration	[31]
QTRAP 5500+	Enzymatic treatment + SPE with ABS Elut NEXUS	Single RP column	500	Not specified	CV 1–24%	SRM validation	Reagent blanks, Matrix blanks, HHEAR QC pools and SRMs	[32]
TSQ Vantage	LLE	RP-LC	200–250	31–263%	<25%	Recovery 71–110% EC No 657/2002	Matrix-matched standards Spike recoveries	[11]
Q Exactive HF-X	Protein precipitation with ACN + 0.5% citric acid followed by Phospholipid removal with HybridSPE	BEH C18 column	100–200	Negligible after cleanup Median 91–107%	<25%	Recovery, precision, matrix effects	Diuron-d6 as performance monitor Pooled plasma reference and ISTD	[34]
Q Exactive Orbitrap HF-X	Phospholipid removal protocol	UHPLC with DIA and DDA Acquity BEH C18 column	50–200	Not specified	Not specified	Reference standardization	Pooled plasma reference	[35]
Q-TOF 6546	Protein precipitation + freeze-drying	2D-LC	90	Variable	Variable <15%	92% detection at 50 ng/mL Spike recoveries of 15 representative standards (70–110%)	Spiked standards	[37]
QTRAP 7500	LLE with Ethyl acetate & hexane	Luna Omega PS C18	200	74–119	<20%	Spike recovery validation	Blanks and Pools	[38]
Triple Quad G6470A	Large volume injection with column switching, Online SPE with custom trap column	RP + mixed-mode ion exchange columns	900	70–130	1.3–18.6%	Spike recovery validation	Isotope standards	[39]

Abbreviations: 2D-LC, Two-Dimensional Liquid Chromatography; ACN, acetonitrile; BEH, bridged ethyl hybrid; CV, coefficient of variation; DBS, dried blood spots; DDA, data-dependent acquisition; DIA, data-independent acquisition; EC, European Commission; GC, Gas Chromatography; G-EQUAS, German External Quality Assessment Scheme; HHEAR, Human Health Exposure Analysis Resource; HILIC, Hydrophilic Interaction Liquid Chromatography; HLB, hydrophilic-lipophilic balance; HRMS, High-Resolution Mass Spectrometry; HSS, high strength silica; ICH, International Conference on Harmonization; IS, Internal Standard; LC, Liquid Chromatography; LLE, Liquid–Liquid Extraction; LOD, limit of detection; LOQ, limit of quantification; MeOH, methanol; NH4F, ammonium fluoride; OSEQAS, Organic Substances in Urine Quality Assessment Scheme; PES, Passive Equilibrium Sampling; PFP, Pentafluorophenyl; QC, quality control; QTrap, quadrupole ion trap; RP, reversed phase; RSD, relative standard deviation; SPE, Solid-Phase Extraction; SRM, standard reference material; SSE, signal suppression/enhancement; SST, systems suitability test; UHPLC, ultra-high performance liquid chromatography.

**Table 3:** Chemical Classes, Target Analytes, and Detection Performance Across Different Human Biological Matrices.

Chemical Class	Representative Analytes	Matrix	LOD Range (ng/mL)	LOQ Range (ng/mL)	Detection Frequency (%)	Recovery Range (%)	Concentration Range (ng/mL)	Study
Bile Acids	GCDCA, GUDCA, 3-keto-LCA	CSF	Not specified	Not specified	Variable	Not specified	Variable 0–1552 nM	[36]
Bisphenols	BPA, BPS	Hair	0.57–5.47 pg/mg	Not specified	100%	Not specified	2.81–35.9 pg/mg median	[33]
Bisphenols	BPA, BPS, BPF	Multiple matrices	0.2–1.5	0.5–5	Variable	76–108%	ND-1.6	[11]
Bisphenols	BPA, BPS, BPF	CSF, Serum	0.01–0.31	Not specified	19–54%	72–128%	<LOD-44	[38]
CEC’s	PFAS, Pesticides, Industrial waste, Personal care, Alkaloids, Sweeteners	Water, urine, Sewage waste	Not Specified	≤10 ng/L Majority being ≤1 ng/L	Variable	80–120%	Total CEC concentration 1.1 × 10^3^–8.8 × 10^3^ ng/L	[39]
Environmental Phenols	BPA, BPS, Triclosan, BP3	Urine	0.01–1.0 majority < 0.5	0.1–3	Variable	83–109	0.00–11.36	[12]
Flame Retardants	DBUP, DPHP, BCPP	Human urine	0.01–1.0	Not specified	≥20%	Not specified	Variable	[29]
Mixed Chemicals	>400 organic chemicals	Human plasma	Not specified	Not specified	Not specified	Mean chemical recoveries 35–62%	10–320 ng/mL	[19]
Mycotoxins	Aflatoxins B1, B2, G1, G2	DBS	0.01–0.1 ng/mL	Not specified	Not specified	60–140%	Not specified	[26]
Mycotoxins	ZEN, α-ZEL, β-ZEL	Breast milk, Urine	0.05–45	0.15–140	Variable Majority ND	71–110%	Variable, Mostly <LOQ	[11]
Organophosphate	DMP, DMTP, PNP	Hair	0.01–3.14 pg/mg	Not specified	93–100%	Not specified	0.15–10 pg/mg Median	[33]
Organophosphate esters	DPHP, BDCIPP	Plasma	Not specified	0.002–6.9	>70%	Spike recovery extraction efficiency 50–150%	3.06 median	[28]
Organophosphate Esters	DPHP, BCPP	Urine	0.01–1.0 majority < 0.5	0.2–2	Variable	83–109	0.00–4.17	[12]
PAHs	NAP1, NAP2	Urine	0.01–1.0 majority < 0.5	0.1–2	Variable	83–109	0.00–768	[12]
PAHs	NAP1, PHEN3, FLUO2	Human urine	0.01–1.0	Not specified	≥20%	Not specified	Variable	[29]
Parabens	Methylparaben, propylparaben	Urine, follicular fluid	0.004–0.53	0.012–1.069	-	62–137	ND-256	[31]
Parabens	Methylparaben, ethylparaben, propylparaben	Urine/Serum/Breast milk	0.02–0.3	0.08–1	Variable	77–110%	Variable ND-28.3	[11]
Pesticides	Organophosphates, pyrethroids	Urine	0.0003–6.3	0.0008–19	Not specified	81–120%	Not specified	[27]
Pesticides	2,4-D, TCP, trans-DCCA	Urine	0.01–1.0 majority < 0.5	0.02–3	Variable	83–109	0.00–24.6	[12]
Pesticides	PBA, CINA6, CDCCA, DMDP, DMTP, PNP	Human urine	0.01–1.0	Not specified	≥20%	Not specified	Variable	[29]
Pesticides	Chlorothalonil-4-hydroxy	Plasma	Not specified	Not specified	100	Not specified	1.1–11.5 (Representative analyte)	[35]
Pesticides	Various classes	Serum	Not specified	Not specified	Variable	Variable	Variable, At least 58 residues detected	[37]
PFAS	PFOA, PFOS, PFHxS	Urine, plasma, serum	0.015–50 pg/mL	Not specified	Up to 67%	60–130	Variable	[24]
PFAS	PFOA, PFOS, PFHxS, PFDA	Plasma	Not specified	0.005–0.281	>70%	89.6–110.2% (NIST recovery) Spike recovery extraction efficiency 50–150%	8.28 median	[28]
PFAS	PFOA, PFOS	Plasma, breast milk	Mostly < 0.05	0.016–0.22	86–100	Median 54–93	<0.092–12	[16]
PFAS	PFBA, PFOA, PFHxS, PFOS	Follicular fluid, seminal fluid	0.005–0.319	0.01–0.728	Variable	62–137%	Major representatives ND-2.91	[31]
PFAS	PFOS, PFOA, PFHxS, PFNA	Plasma	Not specified	0.02–0.16 ng/mL	Variable More than 50% analytes have DF > 70%	77–87%	Variable ND-11.3	[34]
PFAS	PFOA, PFBS, PFHxS	CSF, Serum	0.001–0.02	Not specified	18–100%	72–128%	<LOD-91.3 [Serum] <LOD-2.03 [CSF]	[38]
Phenolic compounds	OH-PAHs, BRPs, OH-PBDEs	Urine	0.008–0.161	Not specified	Variable Majority > 70%	52.5–143%	Total 2.37–117	[30]
Phthalates	MMP, MEHP, MIBP	Human urine	0.01–1.0	Not specified	≥20%	Not specified	Variable	[29]
Phthalates	MEHP, MBP, DEHP metabolites	Urine	0.078–0.425	0.236–1.289	0–100%, Majority > 90%	85–114%	ND (min) > 200 (max) Mean: ND-27.8	[13]
Phthalates	DEHP metabolites, DiNP metabolites	Urine	0.004–0.53	Not specified	6–100%	70–129%	<LOD-33.2	[32]
Phytoestrogens	EQU, ETL	Human urine	0.01–1.0	Not specified	≥20%	Not specified	Variable	[29]
Synthetic antioxidants	BHT-CHO, BHT-COOH, DPG	Plasma	Not specified	0.006–4.9	>70%	Spike recovery extraction efficiency 50–150%	29.1 median	[28]
Synthetic Phenolic Antioxidants	AO2246, 4-tOP, BHA, BHT	Serum	Not specified	Method LOQ 0.034–24.1 ng/g	>93%	36–125% Average 73%	1.4–520 ng/g	[20]
Veterinary Drugs	β-lactams, tetracyclines, sulfonamides	Urine	0.009–3.8 ng/mL	0.027–12.6 ng/mL	Not specified	81–120%	Not specified	[27]
Veterinary drugs	Various classes	Serum	Not specified	Not specified	Variable	Variable	Variable At least 58 residues detected	[37]
VOCs	HEMA2, AAMA, CEMA, HPMA	Human urine	0.01–1.0	Not specified	≥20%	Not specified	Variable	[29]

Abbreviations: 2,4-D, 2,4-dichlorophenoxyacetic acid; 3-keto-LCA, 3-keto-lithocholic acid; 4-tOP, 4-tert octylphenol; AAMA, N-acetyl-S-(2-carbamoylethyl)-L-cysteine; AO2246, 2,2′-methylenebis(4-methyl-6-tert-butylphenol); BCPP, bis(1-chloro-2-propyl) phosphate; BDCIPP, Bis(1,3-dichloro-2-propyl) Phosphate; BHA, butylated hydroxyanisole; BHT, butylated Hydroxytoluene; BP-3, benzophenone-3; BPA, bisphenol A; BPB, bisphenol B; BPS, bisphenol S; BPF, bisphenol F; BRPs, Brominated Phenols; CDCCA, cis-3-(2,2-dichlorovinyl)-2,2-dimethyl-cyclopropane-1-carboxylic acid; CEMA, N-acetyl-S-(2-carboxyethyl)-L-cysteine; CINA6, 6-chloronicotinic acid; DBUP, dibutyl phosphate; DEHP, di-2-ethylhexyl phthalate; DiNP, di-iso-nonyl phthalate; DMDP, Dimethyldithiophosphate; DMTP, Dimethylthiophosphate; DOCP, diphenyl cresyl phosphate; DPCP, diphenyl phenyl phosphate; DPG, 1,3-Diphenylguanidine; DPHP, diphenyl Phosphate; ETL, Enterolactone; EQU, Equol; FLUO2, 2-hydroxyfluorene; GCDCA, glycochenodeoxycholic acid; GUDCA, glycoursodeoxycholic acid; HEMA2, N-acetyl-S-(2-hydroxyethyl)cysteine; HPMA, N-acetyl-S-(3-hydroxypropyl)-L-cysteine; LOD, limit of detection; LOQ, limit of quantification; MBP, mono-n-butyl phthalate; MEHP, mono-2-ethylhexyl phthalate; MEOHP, mono-2-ethyl-5-oxohexyl phthalate; MIBP, mono-isobutyl phthalate; MMP, Monomethyl phthalate; NAP1, 1-naphthol; NAP2, 2-naphthol; OH-PAHs: Hydroxyl Polycyclic Aromatic Hydrocarbons; OH-PBDEs, Hydroxyl Polybrominated Diphenyl Ethers; PAHs, polycyclic aromatic hydrocarbons; PBA, 3-Phenoxybenzoic acid; PFAS, per- and polyfluoroalkyl substances; PFBA, perfluorobutanoic acid; PFBS, perfluorobutanesulfonic acid; PFDA, Perfluorodecanoic Acid; PFOA, perfluorooctanoic acid; PFOS, perfluorooctanesulfonic acid; PFHxS, perfluorohexanesulfonic acid; PFNA, perfluorononanoate; PHEN3, 3-hydroxyphenanthrene; PNP, 4-Nitrophenolperfluorononanoate; TCIPP, tris(2-chloroisopropyl) phosphate; TCP, trichloro-pyridinol; Trans-DCCA, trans-3-(2,2-dichlorovinyl)-2,2-dimethylcyclopropane carboxylic acid; TEP, triethyl phosphate; TPHP, triphenyl phosphate; VOCs, Volatile Organic Compounds; ZEN, Zearalenone.

## Data Availability

Not applicable.

## Abbreviations

The following abbreviations are used in this manuscript:

2,4-D	2,4-dichlorophenoxyacetic acid
2D-LC	Two-Dimensional Liquid Chromatography
2D-LC-HRMS	Two-Dimensional Liquid Chromatography-High-Resolution Mass Spectrometry
3-keto-LCA	3-keto-lithocholic acid
4-tOP	4-tert octylphenol
AAMA	N-acetyl-S-(2-carbamoylethyl)-L-cysteine
ACN	Acetonitrile
AO2246	2,2′-methylenebis(4-methyl-6-tert-butylphenol)
BCPP	Bis(1-chloro-2-propyl) phosphate
BDCIPP	Bis(1,3-dichloro-2-propyl) Phosphate
BEH	Bridged ethyl hybrid
BHA	Butylated hydroxyanisole
BHT	Butylated Hydroxytoluene
BP-3	Benzophenone-3
BPA	Bisphenol A
BPB	Bisphenol B
BPS	Bisphenol S
BPF	Bisphenol F
BRPs	Brominated Phenols
CDCCA	Cis-3-(2,2-dichlorovinyl)-2,2-dimethyl-cyclopropane-1-carboxylic acid
CECs	Contaminants of Emerging Concern
CEMA	N-acetyl-S-(2-carboxyethyl)-L-cysteine
CINA6	6-chloronicotinic acid
CSF	Cerebrospinal fluid
CV	Coefficient of variation
DBUP	Dibutyl phosphate
DBS	Dried blood spots
DDA	Data-dependent acquisition
DEHP	Di-2-ethylhexyl phthalate
DIA	Data-independent acquisition
DiNP	Di-iso-nonyl phthalate
DMDP	Dimethyldithiophosphate
DMTP	Dimethylthiophosphate
DOCP	Diphenyl cresyl phosphate
DPCP	Diphenyl phenyl phosphate
DPG	1,3-Diphenylguanidine
DPHP	Diphenyl Phosphate
EC	European Commission
ECHO	Environmental influences on Child Health Outcomes
EPOLs	Environmental Pollutants
ETL	Enterolactone
EQU	Equol
FLUO2	2-hydroxyfluorene
GC	Gas Chromatography
GC-MS	Gas chromatography-mass spectrometry
GC-HRMS	Gas Chromatography-High-Resolution Mass Spectrometry
GCDCA	Glycochenodeoxycholic acid
GDM	Gestational Diabetes Mellitus
G-EQUAS	German External Quality Assessment Scheme
GUDCA	Glycoursodeoxycholic acid
HEMA2	N-acetyl-S-(2-hydroxyethyl)cysteine
HHEAR	Human Health Exposure Analysis Resource
HILIC	Hydrophilic Interaction Liquid Chromatography
HLB	Hydrophilic-lipophilic balance
HPMA	N-acetyl-S-(3-hydroxypropyl)-L-cysteine
HRMS	High-Resolution Mass Spectrometry
HSS	High strength silica
ICH	International Conference on Harmonization
IS	Internal Standard
LC	Liquid Chromatography
LLE	Liquid–Liquid Extraction
LC-ESI-MS/MS	Liquid Chromatography-Electrospray Ionization-Tandem Mass Spectrometry
LC-HRMS	Liquid Chromatography-High-Resolution Mass Spectrometry
LC-MS/MS	Liquid Chromatography-Tandem Mass Spectrometry
LOD	Limit of detection
LOQ	Limit of quantification
MBP	Mono-n-butyl phthalate
MEHP	Mono-2-ethylhexyl phthalate
MeOH	Methanol
MEOHP	Mono-2-ethyl-5-oxohexyl phthalate
MIBP	Mono-isobutyl phthalate
MMP	Monomethyl phthalate
NAP1	1-naphthol
NAP2	2-naphthol
NH4F	Ammonium fluoride
OH-PAHs	Hydroxyl Polycyclic Aromatic Hydrocarbons
OH-PBDEs	Hydroxyl Polybrominated Diphenyl Ethers
OSEQAS	Organic Substances in Urine Quality Assessment Scheme
PAHs	Polycyclic aromatic hydrocarbons
PBA	3-Phenoxybenzoic acid
PES	Passive Equilibrium Sampling
PFP	Pentafluorophenyl
PFAS	Per- and polyfluoroalkyl substances
PFBA	Perfluorobutanoic acid
PFBS	Perfluorobutanesulfonic acid
PFDA	Perfluorodecanoic Acid
PFOA	Perfluorooctanoic acid
PFOS	Perfluorooctanesulfonic acid
PFHxS	Perfluorohexanesulfonic acid
PFNA	Perfluorononanoate
PHEN3	3-hydroxyphenanthrene
PNP	4-Nitrophenolperfluorononanoate
QC	Quality control
QTrap	Quadrupole ion trap
RP	Reversed phase
RSD	Relative standard deviation
SPE	Solid-phase extraction
SRM	Standard Reference Material
SSE	Signal suppression/enhancement
SST	Systems suitability test
SWATH	Sequential window acquisition of all theoretical fragments
TCIPP	Tris(2-chloroisopropyl) phosphate
TCP	Trichloro-pyridinol
Trans-DCCA	Trans-3-(2,2-dichlorovinyl)-2,2-dimethylcyclopropane carboxylic acid
TEP	Triethyl phosphate
TPHP	Triphenyl phosphate
UHPLC	Ultra-high performance liquid chromatography
VOCs	Volatile Organic Compounds
ZEN	Zearalenone
